# Global burden and influencing factors of chronic kidney disease due to type 2 diabetes in adults aged 20–59 years, 1990–2019

**DOI:** 10.1038/s41598-023-47091-y

**Published:** 2023-11-19

**Authors:** Dandan Xie, Tianpeng Ma, Haoliang Cui, Jing Li, Aihua Zhang, Zhifeng Sheng, Yiqiang Xie

**Affiliations:** 1 College of Traditional Chinese Medicine, International Advanced Functional Omics Platform, Scientific Experiment Center, Hainan Medical University, No. 3, Xueyuan Road, Haikou, 571199 Hainan China; 2 National Clinical Research Center for Metabolic Diseases, Hunan Provincial Key Laboratory of Metabolic Bone Diseases, Health Management Center and Department of Metabolism and Endocrinology, The Second Xiangya Hospital of Central South University, No. 139, Renmin Road, Changsha, 410011 Hunan China; 3 Department of Clinical Nutrition, The First Affiliated Hospital of Hainan Medical University, No. 31, Longhua Road, Haikou, 570102 Hainan China; 4 School of Public Health, Peking University, No. 38, Xueyuan Road, Haidian District, Beijing, 100191 China

**Keywords:** Endocrinology, Health care, Medical research, Nephrology, Risk factors

## Abstract

Population structure and lifestyles may have contributed to the epidemiological status of Chronic Kidney Disease due to Type 2 Diabetes (CKD-T2D). This study is a secondary data analysis. Using data from the Global Burden of Disease Study, we describe the changes in CKD-T2D burden and its influencing factors in the population aged 20–59 years from 1990 to 2019. Globally, the incidence, death, and Disability Adjusted Life Years (DALYs) rate of CKD-T2D showed an upward trend and increased with age, and the burden in males was higher than that in females. Population growth and aging were important driving factors for the increase of CKD-T2D DALY burden, while high systolic blood pressure and high body-mass index were the primary attributable risk factors. High body-mass index exhibited higher contributions to high Socioeconomic Development Index (SDI) countries, whereas low SDI countries were more impacted by high systolic blood pressure. The population attributable fraction of CKD-T2D DALY caused by high body-mass index was positively correlated with SDI, while high temperature and lead exposure were negatively correlated. Therefore, strengthening disease screening for people aged 20–59 years and formulating early intervention measures based on the level of socioeconomic development may effectively alleviate the burden of CKD-T2D.

## Introduction

Diabetic Kidney Disease (DKD) is a prevalent chronic complication of diabetes mellitus, characterized by intermittent or persistent albuminuria and/or progressive decline in the glomerular filtration rate. Without aggressive interventions, DKD ultimately progresses to End-Stage Renal Disease (ESRD)^1^. Type 2 Diabetes (T2D) accounts for over 90% of all types of diabetes, and 40–50% of T2D patients may develop Chronic Kidney Disease (CKD)^2^, a leading cause of mortality in the general population^3,4^. Elderly patients with T2D, especially those with diabetes for more than 10 years, are more likely to progress to CKD than those without T2D^5^. As the population ages, longer life expectancy^6^, and lifestyles change, the incidence of Chronic Kidney Disease due to T2D (CKD-T2D) continues to rise^7,8^ and has become a principal contributor to the global burden of ESRD^9–11^.

Studies have shown that obesity and hypertension are important risk factors for the development and progression of DKD^12–15^. Male sex has also been reported to be a risk factor for the development of DKD^16,17^, and in addition, the incidence and mortality of CKD due to diabetes are closely related to socioeconomic, cultural, and national disease management factors, as well as age^10^. Several studies have reported global and regional trends in incidence, prevalence, and mortality of DKD over time and sex differences across all age groups^18,19^, Pan and Liu et al. focused on the disease burden of diabetes and DKD in China^20,21^, and another study reported mortality and trends in diabetes before 25 years of age^22^. However, few studies have focused on the epidemiological characteristics of people with DKD under the age of 60 years.

Since it usually takes several years for diabetes mellitus to progress to DKD, and the intervention for DKD needs long-term persistence to benefit^23^, the focus should be on early prevention, early recognition, and early intervention within a younger adult demographic. To prevent or delay the disease progression, and further improve the quality of life and survival of the CKD-T2D population^24^, understanding the global burden of CKD-T2D in the population under 60 years old, especially analyzing the factors affecting the disease burden, is particularly important for seeking strategies to prevent and treat DKD and further reduce the incidence rate.

In this study, we focused on describing the global epidemiological characteristics of CKD-T2D in people aged 20–59 years by analyzing the related data from Global Burden of Disease (GBD) 2019, including trends in disease from 1990 to 2019, differences between countries and regions, gender and age, also, we analyzed the driving factors and attributable risk factors for the increase in CKD-T2D Disability Adjusted Life Years (DALYs), as well as the correlation between attributable risk factors and Socio-Demographic Index (SDI).

## Methods

### Data source and measures of burden

The GBD 2019 is a multinational collaborative study that estimates the burden of disease associated with 369 diseases and injuries in 204 countries and territories from 1990 to 2019^25^. The GBD data is obtained for each disease or injury from 7333 national and 24,657 subnational vital registration systems, 16,984 published studies, and 1654 household surveys, as well as other relevant data sources, such as population censuses, health service utilization, satellite imaging, etc^25^. Annual updates include updates to diseases, data sources, and methods, designed to capture annual changes in the same diseases and injuries by age, gender, country, and region using standard epidemiological and health measures such as incidence, prevalence, death rates, and DALYs^25^. DALYs are a commonly used measure of disease burden in epidemiological research, representing the total healthy life years lost by patients from disease onset to death, including years of life lost due to premature death from disease and years of healthy life lost due to disability, and can be expressed as a number or rate^26^.

### Study protocol

This study is a secondary data analysis based on the GBD study. Relevant data, inclusive of CKD-T2D incidence, DALY, and mortality spanning the period 1990 to 2019, was sourced from the Global Health Data Exchange (https://ghdx.healthdata.org/gbd-resultstool). This data was stratified by age, region, and gender. The primary aim of the analysis was to elucidate the global epidemiological characteristics of CKD-T2D, with a specific emphasis on the 20–59 age group. We conducted a decomposition analysis (Supplementary Methods) of age structure, population growth, and epidemiological changes to discern the principal drivers of DALY burden attributable to CKD-T2D among the target population. Furthermore, our investigation sought to identify the primary attributable risk factors contributing to CKD-T2D within diverse SDI categories across regions and countries.

### Disease criteria

The disease in this study is CKD-T2D (ICD-10 code E11.2-E11.29). In the GBD Study 2019, diabetes was defined as a fasting blood glucose concentration of ≥ 126 mg/dL (7 mmol/L) or diabetes treatment reported^27^. CKD-T2D is defined as CKD due to T2D, lasting longer than three months, characterized primarily by urinary albumin/creatinine ratio ≥ 30 mg/g and/or an estimated glomerular filtration rate < 60 mL/min per 1.73 m^2^^28^.

### Country classification

In our study, we classified countries into quintiles according to the SDI. SDI is a composite indicator that quantifies the level of social and demographic development of a country or region based on metrics such as per capita income, average years of education, and the fertility rate among women under age 25. It ranges from 0 to 1, with 0 representing the lowest per capita income, lowest education level, and highest total fertility rate, and 1 representing the highest per capita income, highest education level, and lowest total fertility rate^29^.

### Estimation of attributable risk factors for CKD-T2D

The GBD 2019 study estimated the disease burden attributable to 87 risk (or risk cluster) factors at the global, regional, and national levels^30^. Risk factor exposures were estimated using population-representative survey and surveillance data, spatiotemporal Gaussian process regression models, or DisMod-MR 2.1^30,31^. In this study, we estimated the attributable DALY of CKD-T2D by multiplying the DALY results for each age-sex-location-year with the Population Attributable Fraction (PAF)^30^.

### Statistical analysis

Based on the world standard population reported in the GBD 2019 study, we estimated the Age-Standardized Rates (ASRs, per 100,000 population) of incidence, death, and DALY and their corresponding 95% Uncertainty Intervals (UIs) using the direct standardization method. To assess temporal trends of incidence, death, and DALY of CKD-T2D in the global population aged 20–59 years from 1990 to 2019, we used the Joinpoint Regression Program software (version 4.9.1.0, National Cancer Institute, USA) to calculate the Average Annual Percent Changes (AAPCs) and their corresponding 95% UIs^32^. More detailed information is provided in the Supplementary Methods.

The age-period-cohort model^33^ based on the Poisson distribution can reflect the temporal trend of disease incidence or death across three dimensions: age, period, and cohort. We further fitted the two-factor and three-factor models, including age-period, age-cohort, and period-cohort models for the former, and the APC-IE model^34^ for the latter, and selected the best model to analyze the effects of age, period, and cohort on the incidence and death of CKD-T2D (Supplementary Methods).

Statistical analyses were conducted using R (version 4.2.1) and Stata (version 16) in this study. A *P*-value < 0.05 was considered statistically significant.

### Ethics approval and consent to participate

This research has been conducted using publicly available aggregated data from GBD 2019. As the authors did not collect any new data and only used pre-existing, de-identified data, no additional ethics review board approval was required for this study.

## Results

### Global and regional level

**Figure 1:**
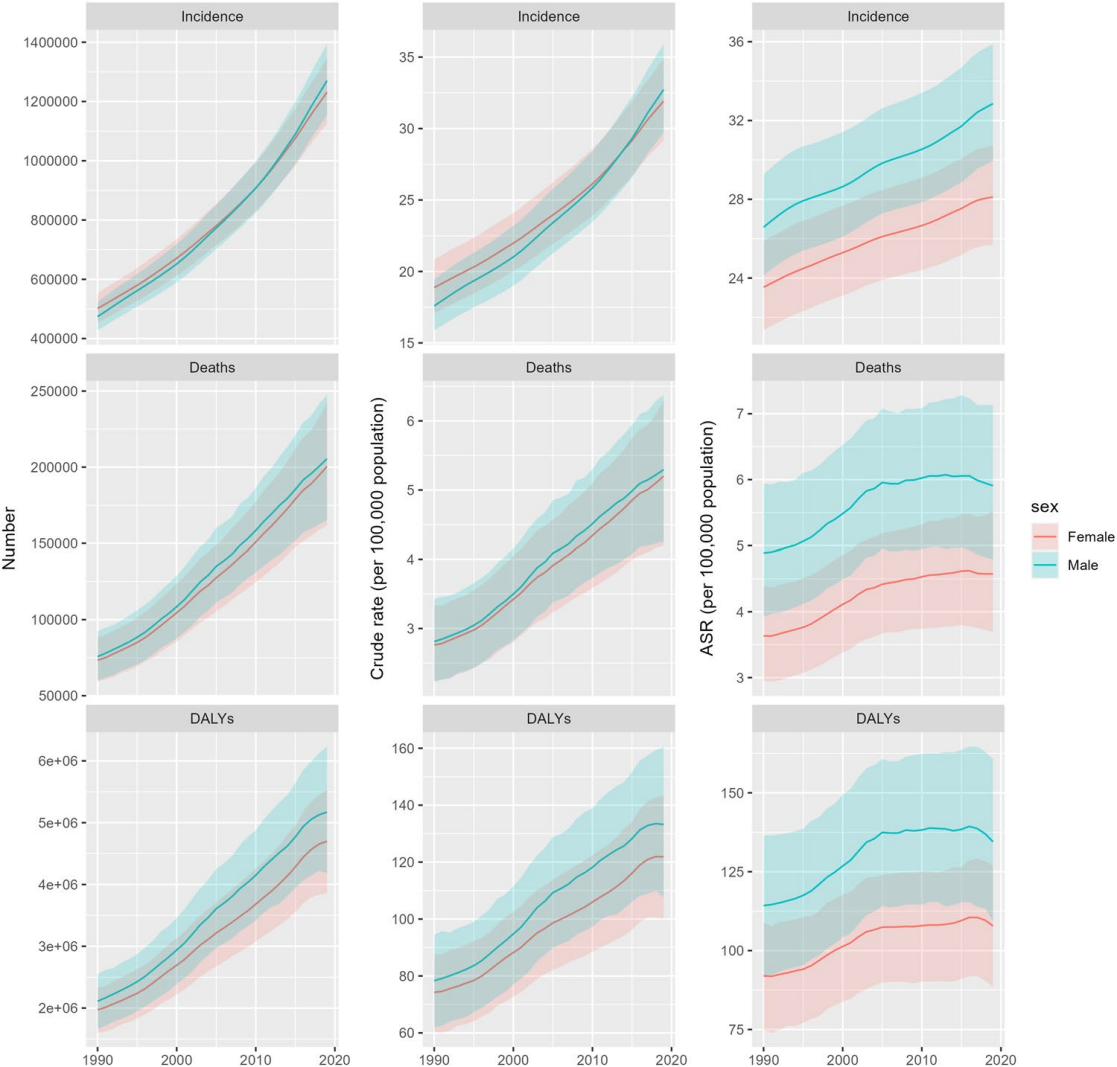
Incidence, deaths, and disability-adjusted life years (DALYs) of chronic kidney disease due to type 2 diabetes (CKD-T2D) from 1990 to 2019: total numbers, crude rates, and age-standardized rates.

In 2019, 2501.2 thousand cases of CKD-T2D were reported globally, with an age-standardized incidence rate of 30.3 per 100,000, a 21.8% increase since 1990. The number of deaths due to CKD-T2D was 406.0 thousand, with an age-standardized death rate of 5.2 per 100,000, an increase of 24.6% since 1990. The number of DALYs was 9870.4 thousand, with an age-standardized rate of 120.2 per 100,000, an 18.2% increase since 1990 (Fig. 1, Supplementary Table S1). The global incidence and death rates of CKD-T2D increased with age, peaking in the 75–79 age group and declining subsequently (Supplementary Fig. S1a), with the highest death rate in the oldest age group (≥ 95 years) (Supplementary Fig. S1b). The trends in crude and age-standardized incidence, death, and DALY rates of CKD-T2D in the population aged 20–59 years in different regions globally between 1990 and 2019 were illustrated in Supplementary Fig. S2.

**Figure 2:**
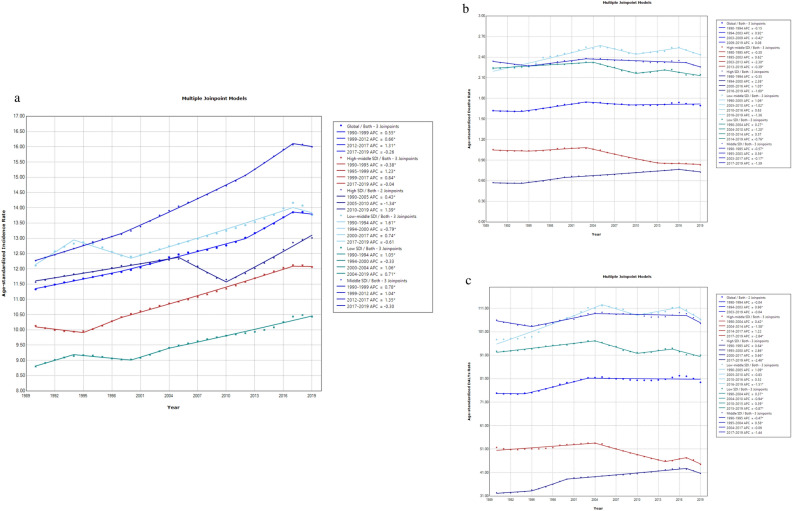
Burden of chronic kidney disease due to type 2 diabetes (CKD-T2D) patients aged 20–59 years, globally and by socio-demographic index (SDI) from 1990 to 2019. (**a**) Age-standardized incidence rate, (**b**) age-standardized deaths rate, (**c**) age-standardized disability adjusted life years (DALYs) rate.

Furthermore, we performed trend analysis using the Joinpoint software. Over the past 30 years, the global age-standardized incidence rate of CKD-T2D in the population aged 20–59 years showed an upward trend (AAPC = 0.7%, *P* < 0.05), with high SDI countries exhibiting greater fluctuations, a significant decline during 2005–2010 [Annual Percent Change, (APC) =  − 1.3%, *P* < 0.05, followed by a significant increase since 2010 (APC = 1.4%, *P* < 0.05) (Fig. 2a). The age-standardized death rate increased slightly (AAPC = 0.2%, *P* < 0.05), with varying degrees of decline observed in different SDI countries since 2016, and the most significant decrease in high SDI countries (APC =  − 1.6%, *P* < 0.05) (Fig. 2b). The age-standardized DALY rate was similar to the death rate (AAPC = 0.3%, *P* < 0.05) (Fig. 2c).

### National level

**Figure 3:**
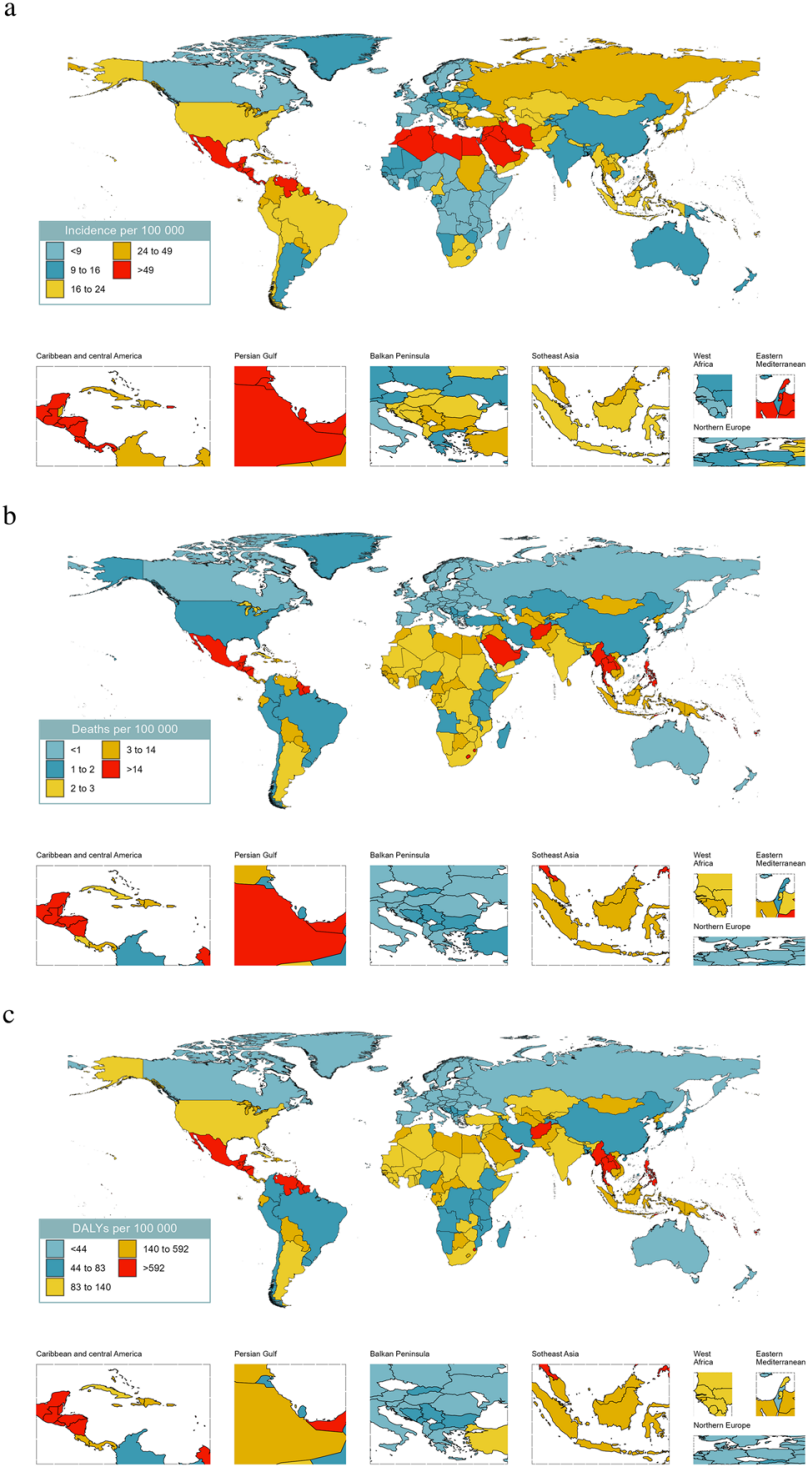
Global burden of chronic kidney disease due to type 2 diabetes (CKD-T2D) patients aged 20–59 years per 100,000 population in 2019. (**a**) Age-standardized incidence, (**b**) age-standardized deaths rate, (**c**) age-standardized disability adjusted life years (DALYs).

In 2019, the age-standardized incidence rate of CKD-T2D in the population aged 20–59 years in 204 countries globally ranged from 5.0 to 49.2 per 100,000, with the highest incidence rate observed in Costa Rica and the lowest in Uganda (Fig. 3a). The age-standardized death rate ranged from 0.1 to 14.2 per 100,000, with the highest in Mauritius (Fig. 3b). The age-standardized DALY rate ranged from 7.1 to 591.8 per 100,000, with the highest in Mauritius and the lowest in Iceland (Fig. 3c).

From 1990 to 2019, the percentage change in the age-standardized incidence rate of CKD-T2D in the population aged 20–59 years demonstrated significant variation across countries, with Bahrain experiencing the largest increase of 133.2%, whereas India (− 4.4%) and Spain (− 3.1%) exhibited contrasting trends (Supplementary Fig. S3a). During the same period, the largest increase in age-standardized death rate occurred in Armenia (388.2%), and the largest decrease was in Maldives (− 64.4%) (Supplementary Fig. S3b). El Salvador (240.7%), Armenia (238.1%), and Mexico (178.2%) were the top three countries with the greatest increase in age-standardized DALY rate, while Maldives (− 59.1%), Ethiopia (− 57.9%), and Poland (− 47.7%) exhibited the most substantial decreases (Supplementary Fig. S3c).

### Age and sex patterns

For the population aged 20–59 years, the number and rate of incidence, death, and DALY all increased with age (Supplementary Fig. S4, Table S2), with similar trends for both sexes, but males bore a higher burden. In the 55–59 age group, the number of deaths (Supplementary Fig. S4b) and DALYs (Supplementary Fig. S4c) were roughly 1.3 times higher for males than for females.

**Figure 4:**
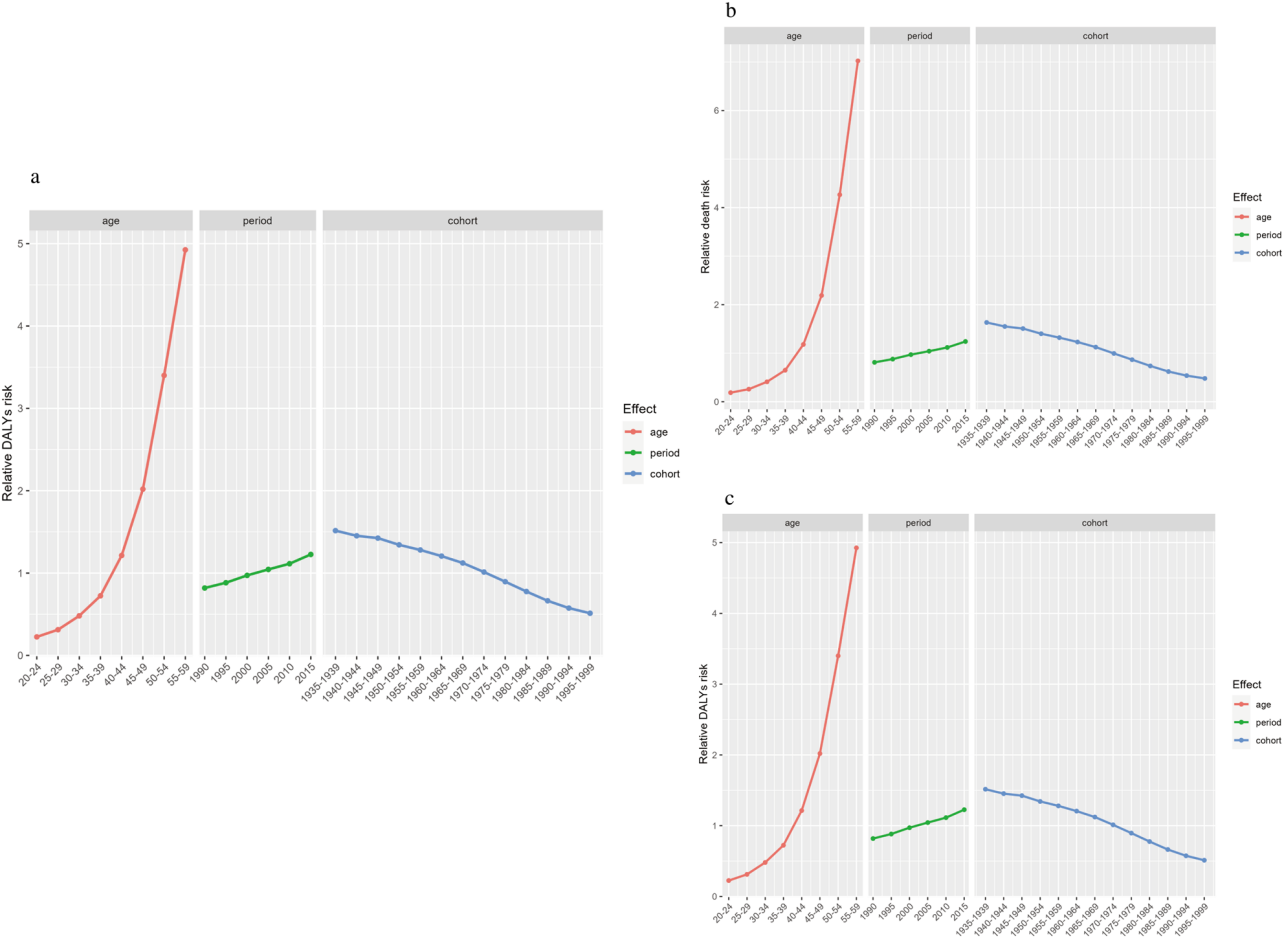
Age, period, and cohort effects on the global relative risk of chronic kidney disease due to type 2 diabetes (CKD-T2D). (**a**) Relative incidence risk, (**b**) relative deaths risk, (**c**) relative disability adjusted life years (DALYs) risk.

The effects of age, period, and cohort on the risk of CKD-T2D incidence, death, and DALYs were further explored (Fig. 4, Supplementary Table S3, Fig. S5). Our findings demonstrated a persistent increase in the risk of CKD-T2D incidence, death, and DALYs with age, even after controlling for period and cohort effects. Specifically, in the 55–59 age group, the Rate Ratio (RR) values for incidence, death, and DALYs were 8.21 (95% CI 8.19–8.24), 7.02 (95% CI 6.98–7.07), and 4.93 (95% CI 4.92–4.93), respectively (Fig. 4, Supplementary Table S3, Fig. S5). The period effects for incidence, death, and DALYs showed a slightly increasing trend from 1990 to 2015 (Fig. 4, Supplementary Table S3, Fig. S5), with the incidence risk increasing from 2005 (RR 1.06, 95% CI 1.06, 1.06) to 2015 (RR 1.46, 95% CI 1.46, 1.47), while the risk of death and DALYs were similar to the incidence during this period. The cohort effects indicated that the later-born cohorts had a lower risk of CKD-T2D incidence, death, and DALYs (Fig. 4, Supplementary Table S3, Fig. S5).

### Drivers of CKD-T2D epidemiology: population growth, aging, and epidemiologic changes

**Figure 5:**
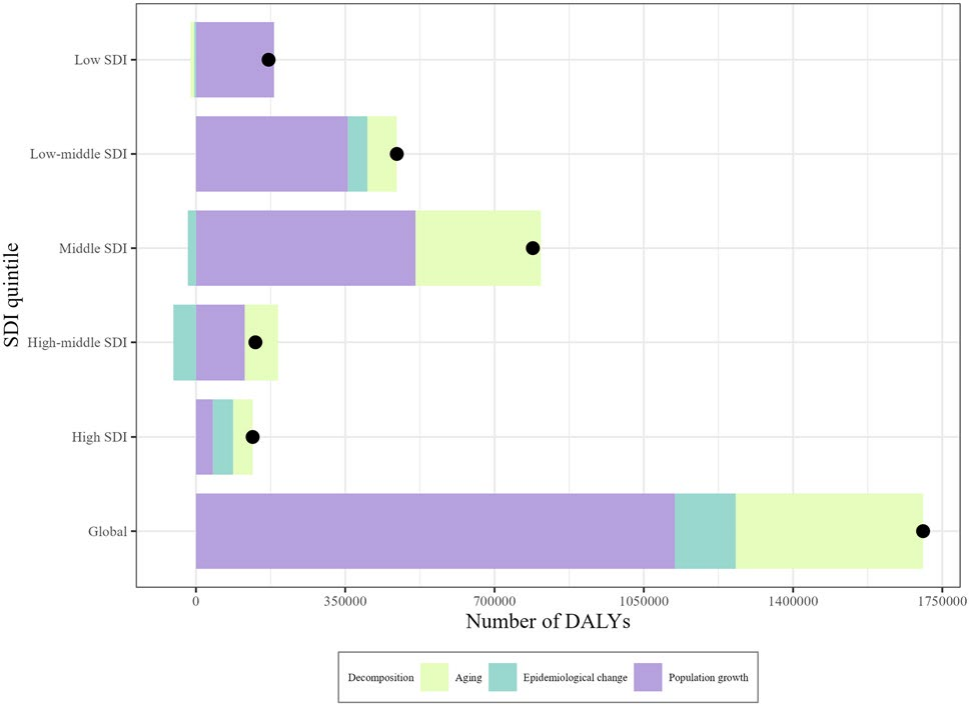
Changes in chronic kidney disease due to type 2 diabetes (CKD-T2D) patients aged 20–59 years disability adjusted life years (DALYs) according to population-level determinants of population growth, aging, and epidemiological change from 1990 to 2019 at the global level and by socio-demographic index (SDI) quintile.

To explore the effects of population growth, aging, and epidemiological changes on the epidemiology of CKD-T2D in the population aged 20–59 years, we performed a decomposition analysis of raw DALYs. Overall, over the past 30 years, CKD-T2D DALY in the 20–59 age group has increased significantly globally, with the most pronounced increase in middle SDI countries (Fig. 5). Population growth and aging were found to be the primary contributors to the CKD-T2D DALY burden globally, accounting for 65.9% and 25.8%, respectively (Supplementary Fig. S6, Table S4). In middle SDI (65.2%), low-middle SDI (75.7%), low SDI (107.5%), and high-middle SDI (82%) countries, population growth was the key driver of the increase in CKD-T2D DALY, whereas, in high SDI countries, the contributions of population growth, aging, and epidemiological changes to DALY increase were relatively consistent. The epidemiological changes, which reflect the underlying changes in age and population-adjusted CKD-T2D incidence and death rates over the past 30 years, have declined in high-middle SDI, middle SDI, and low SDI countries while aging has only declined in low SDI countries (Fig. 5, Supplementary Fig. S6, Table S4).

### Attributable risk factors for DALY in CKD-T2D

**Figure 6:**
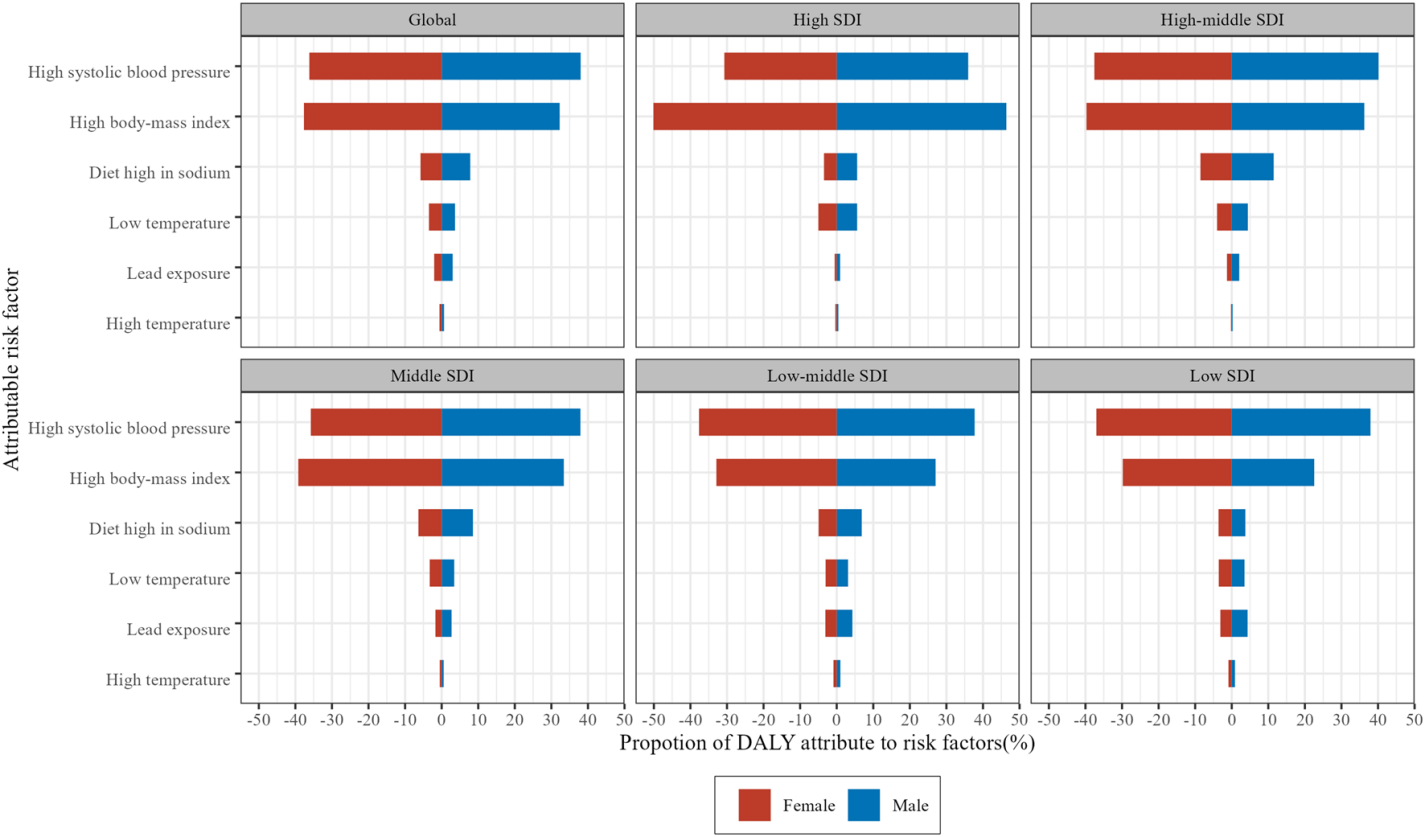
Proportion of chronic kidney disease due to type 2 diabetes (CKD-T2D) patients aged 20–59 years disability adjusted life years (DALYs) attributable to 6 risk factors in 2019 at the global level and socio-demographic index (SDI) quintile by sex.

The GBD2019 study attributed CKD-T2D DALY to six risk factors across three primary categories, as outlined in Supplementary Table S5. Overall, globally, the DALY for CKD-T2D in the population aged 20–59 years showed a decreasing trend attributed to diet high in sodium, low temperature, and lead exposure over the past 30 years, while attributed to high systolic blood pressure, high body-mass index (BMI), and high temperature showed an increasing trend (Supplementary Fig. S7). In 2019, the top two attributable risk factors for CKD-T2D DALY globally were high systolic blood pressure (37.2%) and high BMI (34.7%). Notably, CKD-T2D DALY in high SDI countries was more attributed to high BMI, whereas in low SDI countries was more attributed to high systolic blood pressure. Gender differences in the contribution of different risk factors were insignificant across different SDI countries (Fig. 6, Supplementary Fig. S8).

Pearson correlation was conducted to examine the relationship between the DALY for CKD-T2D attributable risk factors and SDI. The results showed that the PAF of DALY due to high BMI was positively associated with SDI (R = 0.62 to 0.65, *P* < 0.001), high temperature (R =  − 0.35 to − 0.38, *P* < 0.001), and lead exposure (R =  − 0.62 to − 0.64, *P* < 0.001) were negatively associated with SDI. Moreover, diet high in sodium showed a positive correlation with SDI in 30–49 years old (R = 0.15 to 0.19, *P* < 0.05), while low temperature was positively correlated with SDI in 45–59 years old (R = 0.17 to 0.27, *P* < 0.05). In contrast, no correlation was observed between high systolic blood pressure and SDI (R = 0.02 to 0.12, *P* > 0.05) (Supplementary Fig. S9).

## Discussion

### Global and regional differences in the burden of CKD-T2D

Disparities in health outcomes and disease burdens are commonly observed across different countries and regions due to variations in social development levels^25,29^. Our findings demonstrate that the age-standardized incidence rate of CKD-T2D among the global population aged 20–59 years is highest in the middle SDI regions, while the age-standardized death and DALY rates are highest in the low-middle SDI regions. Countries categorized as middle or low-middle SDI tend to experience more rapid social development and economic transformation than those with higher SDI^35^. Moreover, countries with lower SDI levels typically exhibit lower levels of social development, economic progress, and effective healthcare coverage for their populations^36^. This may be a significant contributing factor to the increased disease burden of CKD-T2D observed in middle SDI and low-middle SDI regions.

### Sex and age differences in the burden of CKD-T2D between 20 and 59 years old

As individuals age, renal function gradually declines^37,38^, and the prevalence of CKD, including DKD, is significantly higher in the elderly than in the young population^39,40^. The American Diabetes Association consensus conference notes a steady increase in the incidence of DKD and ESRD caused by DKD among middle-aged African Americans, Native Americans, Hispanics, and other populations^41^, with males identified as a significant risk factor for DKD progression^16,17^. Our study findings are consistent with these observations and indicate that age and gender are important factors affecting the burden of CKD-T2D. Therefore, implementing screening and intervention measures for CKD-T2D and its risk factors among the population aged 20–59 years, especially in middle-aged men, may be a crucial public health initiative to mitigate the burden of CKD-T2D.

### Prevalent risk factors of CKD-T2D

Adjusting modifiable risk factors to reduce disease burden is a crucial measure in developing public health policy^42^. Alterations in societal lifestyles have led to an increased number of obese individuals^43^ and a concomitant rise in the prevalence of CKD caused by type 2 diabetes^13,44–47^. DKD has become the primary contributor to the disease burden and medical costs of obese type 2 diabetes patients^48^. The risk of type 2 diabetes patients developing CKD is related to obesity and hypertension, and they often share common risk factors^49–52^. Sodium is an essential mineral for the human body, with the functions of regulating osmotic pressure, blood volume, and vascular smooth muscle contraction, and is important for maintaining normal physiological functions. Nonetheless, long-term excessive sodium intake can lead to obesity and hypertension, which can in turn cause type 2 diabetes and CKD^53–55^. Our findings show that globally, the impact of diet high in sodium on CKD-T2D is decreasing, while high systolic blood pressure and high BMI are increasing. This suggests that people may have gradually become aware of the harm of high-sodium diets and have reduced sodium intake in their daily diets. However, the pathogenesis of hypertension is complex and related to various factors such as genetics, environment, metabolism, and exercise, in addition to long-term high-sodium diets^53,56,57^. Therefore, it may be an important preventive measure to reduce the burden of CKD-T2D by carrying out promotional education and strengthening management and treatment for hypertensive and high BMI patients.

The Influence of the environment on CKD-T2D should not be underestimated. Lead, a toxic heavy metal, can accumulate in various tissues including the kidneys, brain, and bones via the bloodstream with prolonged exposure, posing a hazard to human health^58–60^. Our findings show that a decreasing trend in global CKD-T2D DALY attributable to low temperature and lead exposure and an increasing trend in high temperature among the population aged 20–59 years over the past 30 years. It suggests that with the development of society, environmental governance, and improvements in living standards, progress has been made in reducing lead exposure and addressing low-temperature issues through measures such as reducing the use of tobacco, leaded gasoline, and lead-based coatings^61,62^ as well as the advancement of insulation materials and renewable energy technologies. Nevertheless, the effects of global warming and high temperatures on CKD-T2D necessitate the continuous strengthening of environmental protection measures, such as reducing deforestation, the use of petroleum and coal as fuels, enhancing garbage sorting and treatment, and reducing the use of non-biodegradable plastics to promote better health outcomes.

### Regional difference in attributable risk factors for CKD-T2D

In the present study, we investigated the attributable risk factors for CKD-T2D DALYs in the population aged 20–59 years and found that high systolic blood pressure was the main contributing factor in low SDI countries, while high BMI had a greater impact in high SDI countries. The analysis also revealed a positive association between the PAF of CKD-T2D DALYs due to high BMI and SDI, while high temperature and lead demonstrated a negative correlation. The findings suggest that poor public health infrastructure, limited medical resources, and interventions for hypertension may be responsible for the greater impact of high systolic blood pressure in low SDI countries^63^. In contrast, advanced economic and educational levels in high SDI countries lead to a greater awareness of environmental factors such as high temperature and lead exposure, and proactive and comprehensive measures to mitigate the risks. However, countries with higher SDI may have greater availability of high energy density diets, more convenient transportation, and less physical activity or exercise, which could elevate the risk of high BMI^64^. Moreover, despite hypertension being a well-established risk factor for CKD-T2D disease progression^65^, our study did not observe any association between high systolic blood pressure and SDI. This may be attributed to the intricate interplay of multiple factors such as genetics, environment, diet, physical activity, access to healthcare, and medication supply, which differ greatly between countries with different levels of SDI^66^.

The epidemiology and risk factors of CKD-T2D exhibit significant variability across different regions and countries^25^. In countries with higher SDI, which are characterized by pronounced population aging, interventions should focus on addressing this demographic shift and encouraging childbirth^67^. On the other hand, strong evidence shows that longer sedentary time, lower physical activity, and higher red meat consumption are closely related to obesity and T2D^68,69^, and the promotion and popularization of health-oriented lifestyles should be strengthened to alleviate the disease burden of CKD-T2D caused by risk factors such as high BMI. In contrast, in countries with lower SDI, the burden of disease has shifted from communicable to non-communicable diseases^25^, such as CKD-T2D, however, due to constraints in the level of health care coverage and sanitation, changes in health systems are slower than changes in the epidemiologic spectrum^36^, and the effective coverage index for non-communicable diseases is much lower than that for communicable diseases^70^, interventions should prioritize disease care and treatment, management and improvement of environmental health conditions^27^. Therefore, to effectively mitigate the burden of CKD-T2D, global collaboration should be strengthened, and tailored health intervention policies based on each country’s characteristics.

### Strengths and limitations of this study

We conducted a comprehensive evaluation of the burden of CKD-T2D in the population aged 20–59 using the GBD Study 2019 database, including trends in incidence, mortality, and DALYs at global, regional, and national levels, as well as differences based on age and gender. We further analyzed the impact of underlying driving factors and attributable risk factors. Additionally, we explored the correlation between the PAF of CKD-T2D DALYs linked to attributable risk factors and the SDI. This is the first study of its kind targeting this age group. Our research findings can provide insights for the development of early health prevention policies to alleviate the burden of CKD-T2D globally. However, our study has several limitations. Firstly, the insufficient number of risk factors in the GBD study database hindered a more comprehensive analysis, and several common risk factors, such as physical activity, dietary structure, smoking, and drinking, were not included in the evaluation. Secondly, the absence of suitable disease registration systems in some countries resulted in only estimated numbers of CKD-T2D cases or deaths. Thirdly, discrepancies in the definition of CKD-T2D across data sources, although minimized by the GBD study 2019, still impeded complete bias elimination. In addition, the burden of CKD-T2D varies depending on detection method, diagnosis accuracy, and disease registration, with regions and countries of lower SDI potentially underestimating the disease burden. Lastly, the study’s focus was solely on the population aged 20–59 years, thereby excluding the burden of disease among those 60 years or more.

## Conclusions

CKD-T2D has emerged as a growing global public health concern, especially among adults under 60 years old, with a higher disease burden in males than females. Population growth and aging are significant drivers of the increasing burden of CKD-T2D DALYs, with high BMI and high systolic blood pressure recognized as primary modifiable risk factors. Notably, high BMI is the primary determinant in high SDI countries, while high systolic blood pressure has a greater impact in low SDI countries. Therefore, strengthening disease screening for people aged 20–59, and developing tailored early intervention policies based on socioeconomic development levels, may effectively mitigate the CKD-T2D burden.

### Supplementary Information

Supplementary Legends.

Supplementary Figure S1.

Supplementary Figure S2.

Supplementary Figure S3.

Supplementary Figure S4.

Supplementary Figure S5.

Supplementary Figure S6.

Supplementary Figure S7.

Supplementary Figure S8.

Supplementary Figure S9.

Supplementary Information.

Supplementary Table S1.

Supplementary Table S2.

Supplementary Table S3.

Supplementary Table S4.

Supplementary Table S5.

## Data Availability

Data used in the analyses can be obtained from the Global Health Data Exchange Global Burden of Disease Results Tool (https://ghdx.healthdata.org/gbd-results-tool).

## Abbreviations

AAPC: Average annual percent change
APC: Annual percent change
ASR: Age-standardized rate
CKD-T2D: Chronic kidney disease due to type 2 diabetes
DALY: Disability adjusted life year
DKD: Diabetic kidney disease
ESRD: End-stage renal disease
GBD: Global Burden of Disease
PAF: Population attributable fraction
SDI: Socio-demographic index
UI: Uncertainty interval
