# Supplementary material for: Global burden and influencing factors of chronic kidney disease due to type 2 diabetes in adults aged 20–59 years, 1990–2019
Source: Sci Rep. 2023 Nov 19;13:20234. doi: 10.1038/s41598-023-47091-y (PMC10658077; doi:10.1038/s41598-023-47091-y)
Supplement: Supplementary file 1 — Supplementary Legends. [file 41598_2023_47091_MOESM1_ESM.docx]

**Supplementary Material**

**Supplementary Methods.**

**Table S1** Numbers and ASRs per 100,000 in incidence, deaths, and DALYs for CKD-T2D in 2019, and percentage change from 1990 globally and by SDI quintile.

**Table S2** The average annual percentage change of incidence, death and DALYs in CKD-T2D by age, from 1990 to 2019.

**Table S3** Age, period, and cohort effects on the global incidence, death and DALYs relative risk of CKD-T2D.

**Table S4** Changes in DALYs number according to population-level determinants from 1990 to 2019 globally and by SDI quintile.

**Table S5** Attributable risk factors for DALYs in CKD-T2D.

**Fig. S1** Age distribution of global male and female chronic kidney disease due to type 2 diabetes (CKD-T2D) incidence and deaths rate in 1990 and 2019. (a) incidence rate; (b) deaths rate.

**Fig. S2** Trends for crude rate and age-standardized rate (per 100,000 population) of chronic kidney disease due to type 2 diabetes (CKD-T2D) patients aged 20-59 years.

**Fig. S3** Worldwide percentage change of chronic kidney disease due to type 2 diabetes (CKD-T2D) patients aged 20-59 years per 100,000 population in 2019. (a) age-standardized incidence, (b) age-standardized deaths rate, (c) age-standardized disability adjusted life years (DALYs).

**Fig. S4** Worldwide total number and rate of chronic kidney disease due to type 2 diabetes (CKD-T2D) patients aged 20-59 years, by age and sex in 2019. (a) incidence, (b) deaths, (c) disability adjusted life years (DALYs). Rate is per 100,000 population.

**Fig. S5** Global age-specific tends of chronic kidney disease due to type 2 diabetes (CKD-T2D) in different birth cohorts. (a) incidence; (b) deaths rate; (c) disability adjusted life years (DALYs).

**Fig. S6** Changes in chronic kidney disease due to type 2 diabetes (CKD-T2D) patients aged 20-59 years disability adjusted life years (DALYs) percent according to population-level determinants from 1990 to 2019 at the global level and by socio-demographic index (SDI) quintile.

**Fig. S7** Proportion of disability adjusted life years (DALYs) attribute to 6 risk factors in chronic kidney disease due to type 2 diabetes (CKD-T2D) patients aged 20-59 years from 1990 to 2019 at the global level and by socio-demographic index (SDI) quintile.

**Fig. S8** Proportion of disability adjusted life years (DALYs) attribute to 6 risk factors in chronic kidney disease due to type 2 diabetes (CKD-T2D) patients aged 20-59 years in 2019 at the global level and by socio-demographic index (SDI) quintile.

**Fig. S9** The population attributable fractions (PAFs) of disability adjusted life years (DALYs) in chronic kidney disease due to type 2 diabetes (CKD-T2D) patients aged 20-59 years across 204 countries and territories from low socio-demographic index (SDI) to high SDI in 2019.
