# Supplementary figures and images for: Global burden and influencing factors of chronic kidney disease due to type 2 diabetes in adults aged 20–59 years, 1990–2019
Source: Sci Rep. 2023 Nov 19;13:20234. doi: 10.1038/s41598-023-47091-y (PMC10658077; doi:10.1038/s41598-023-47091-y)

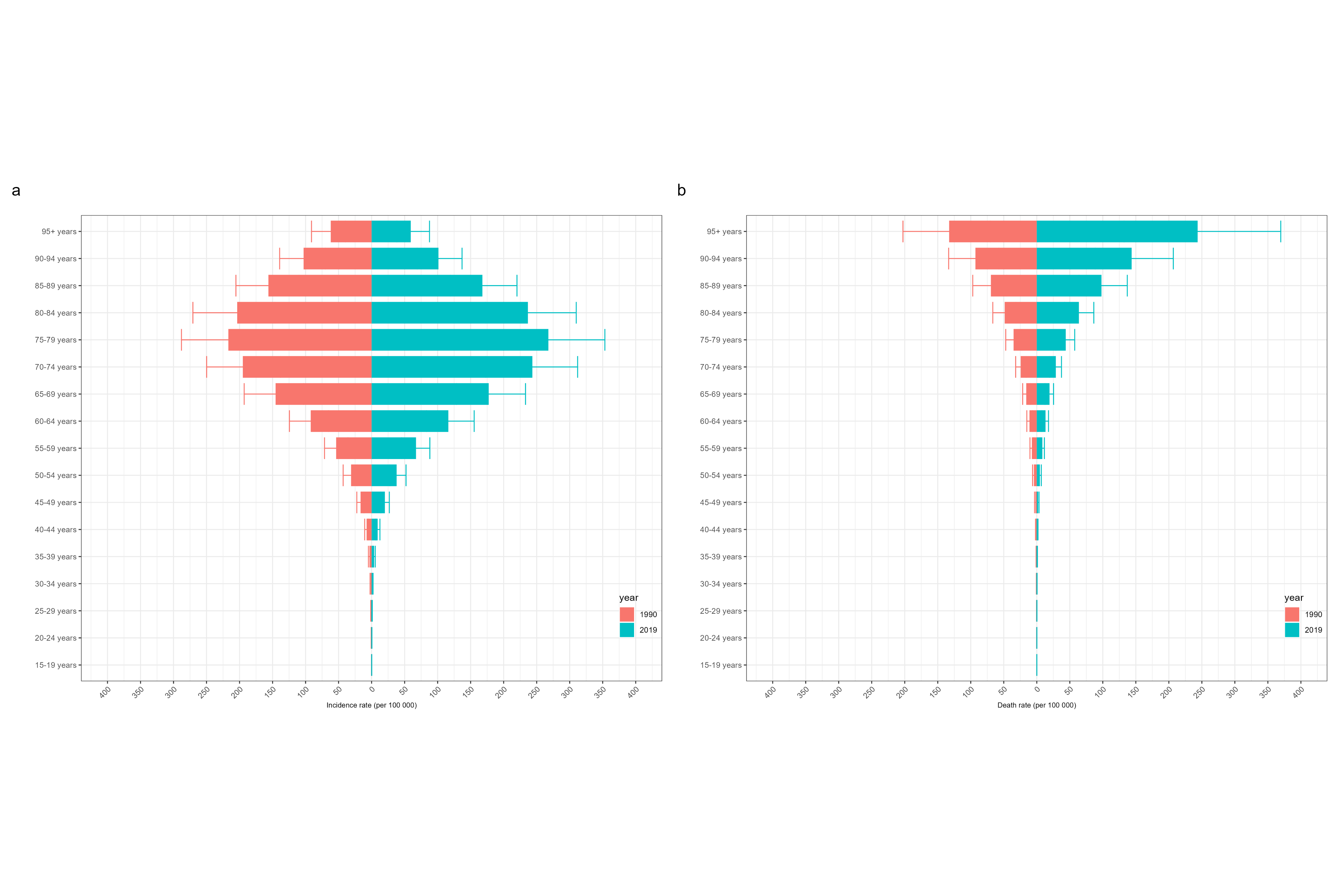

Supplement: Supplementary file 2 — Supplementary Figure S1. [file 41598_2023_47091_MOESM2_ESM.tif]

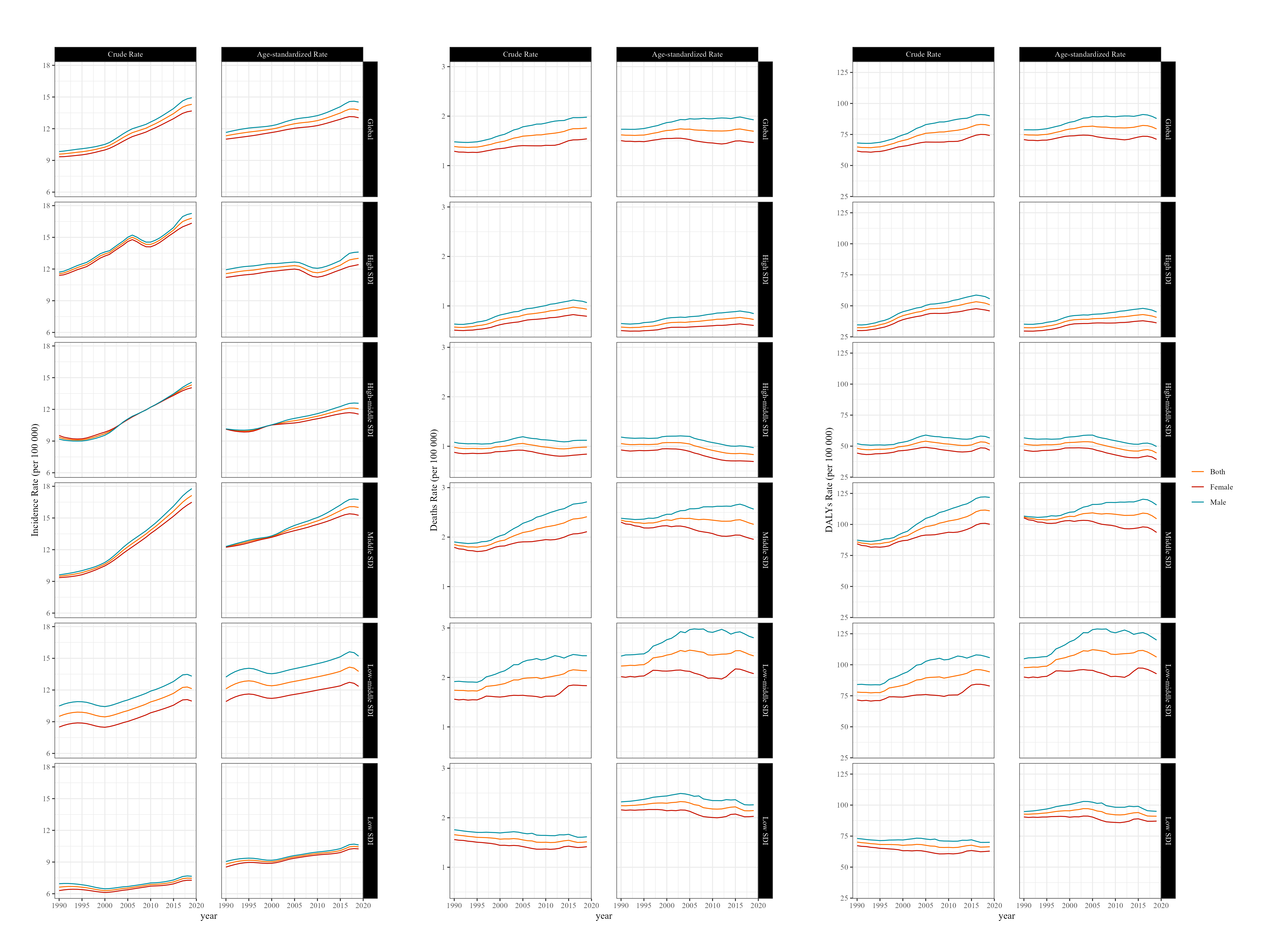

Supplement: Supplementary file 3 — Supplementary Figure S2. [file 41598_2023_47091_MOESM3_ESM.tif]

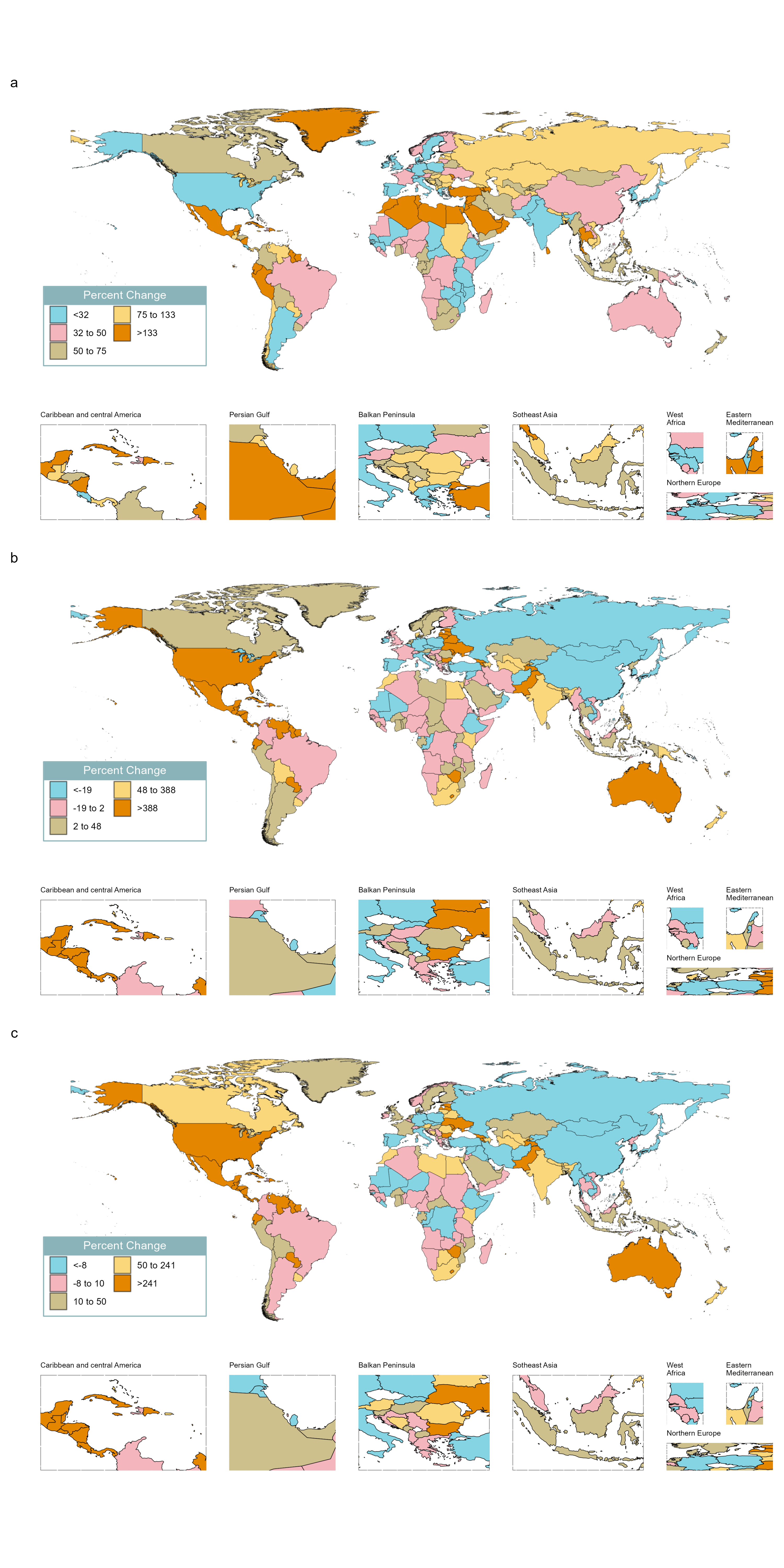

Supplement: Supplementary file 4 — Supplementary Figure S3. [file 41598_2023_47091_MOESM4_ESM.tif]

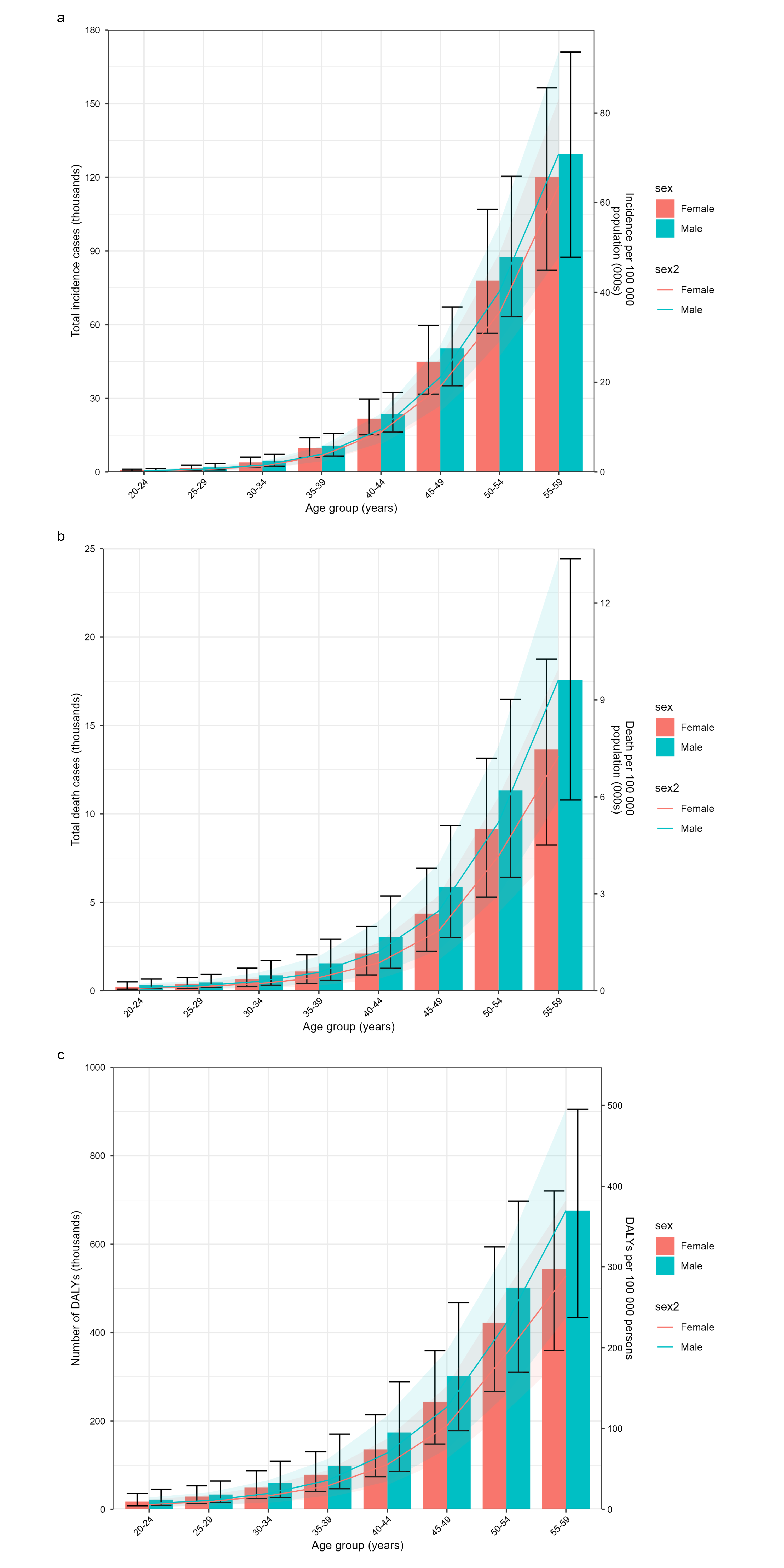

Supplement: Supplementary file 5 — Supplementary Figure S4. [file 41598_2023_47091_MOESM5_ESM.tif]

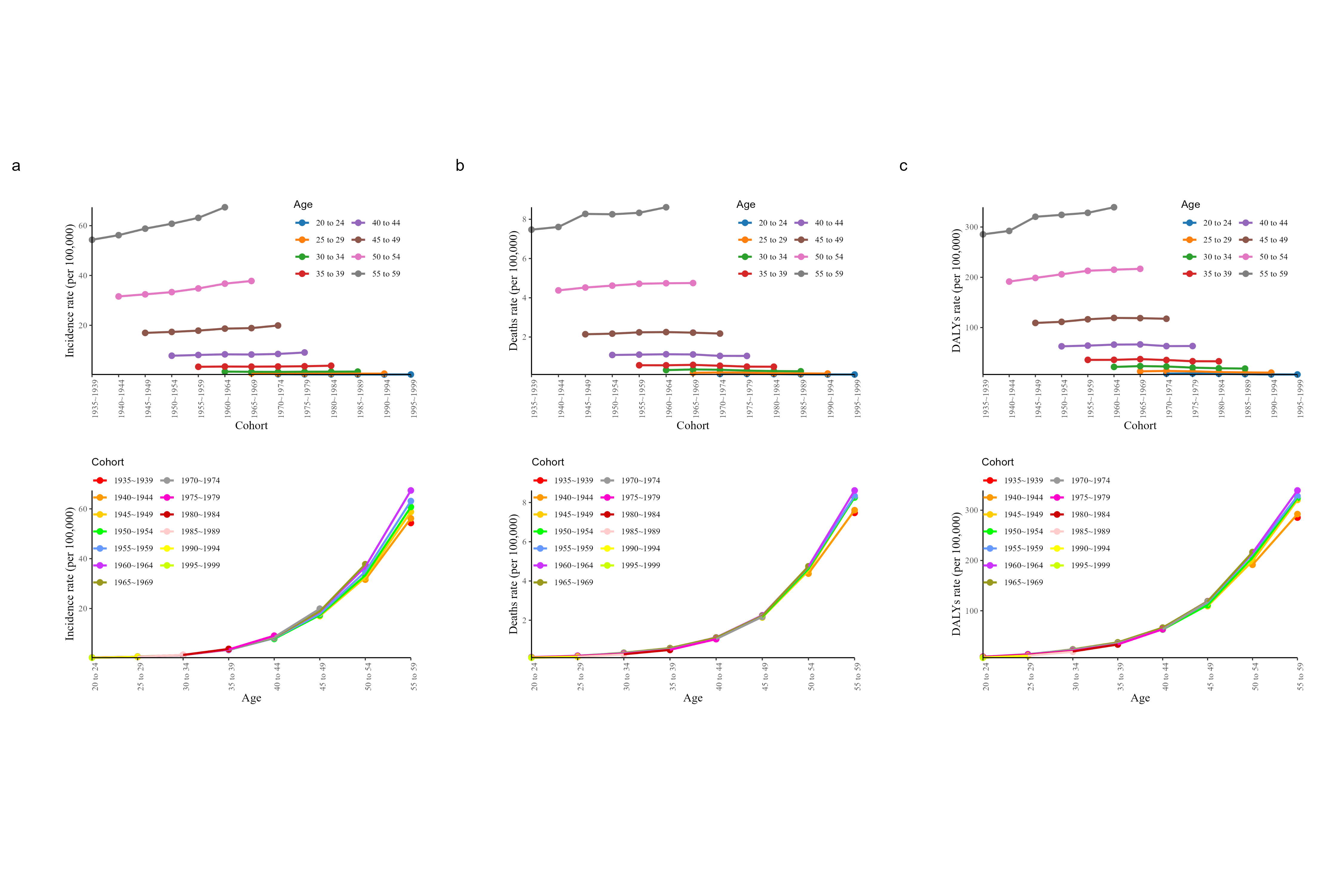

Supplement: Supplementary file 6 — Supplementary Figure S5. [file 41598_2023_47091_MOESM6_ESM.tif]

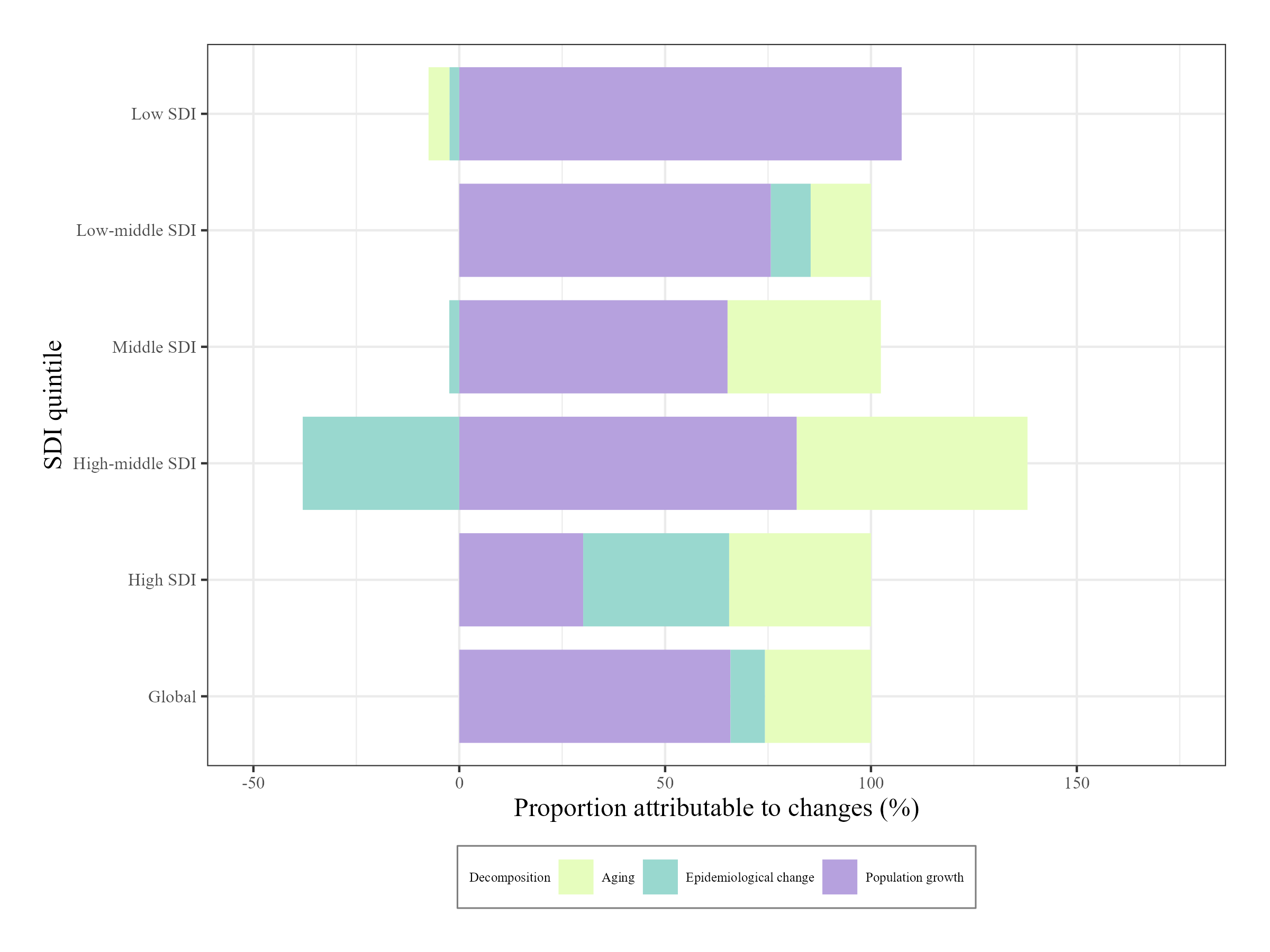

Supplement: Supplementary file 7 — Supplementary Figure S6. [file 41598_2023_47091_MOESM7_ESM.tif]

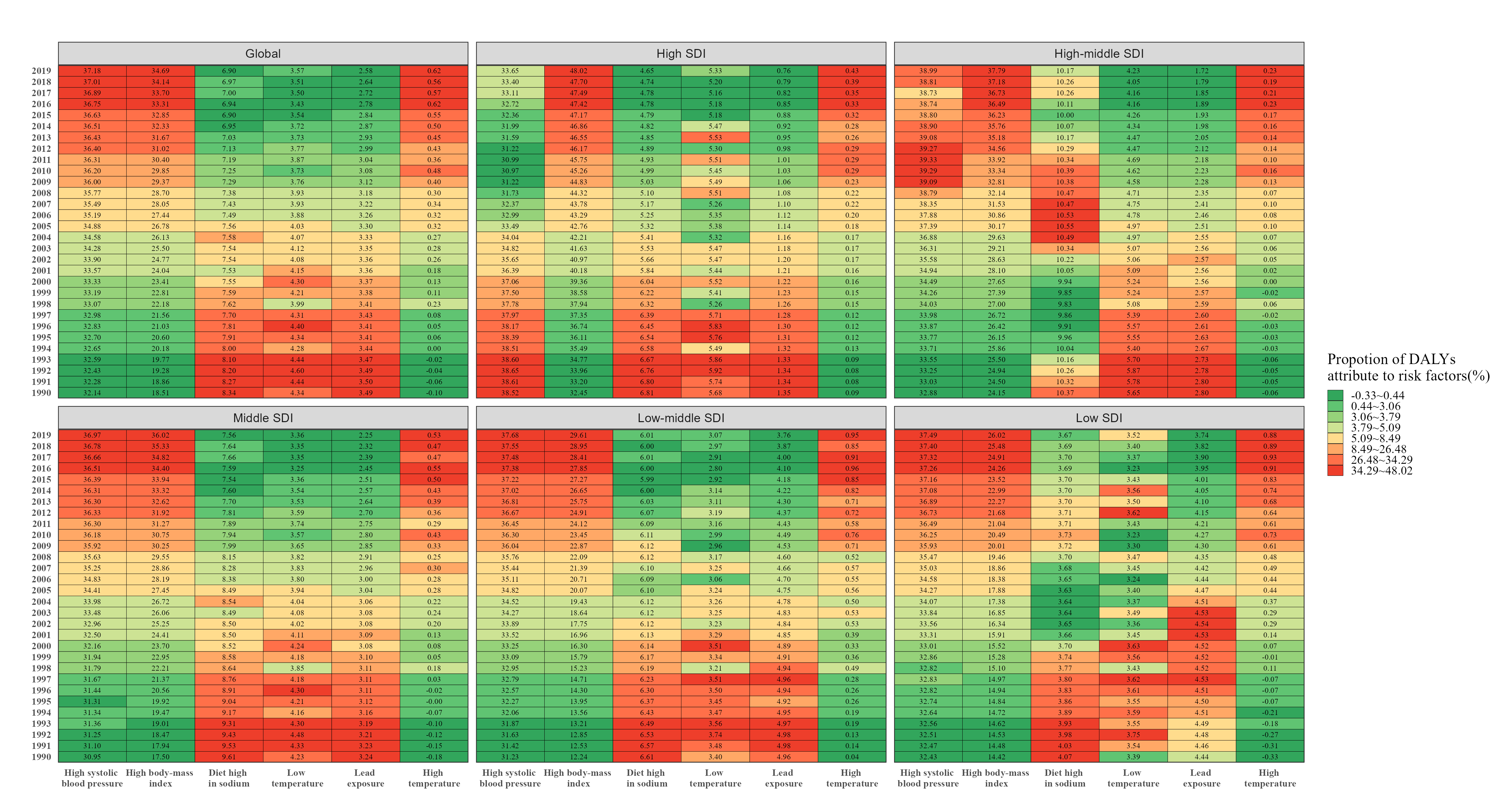

Supplement: Supplementary file 8 — Supplementary Figure S7. [file 41598_2023_47091_MOESM8_ESM.tif]

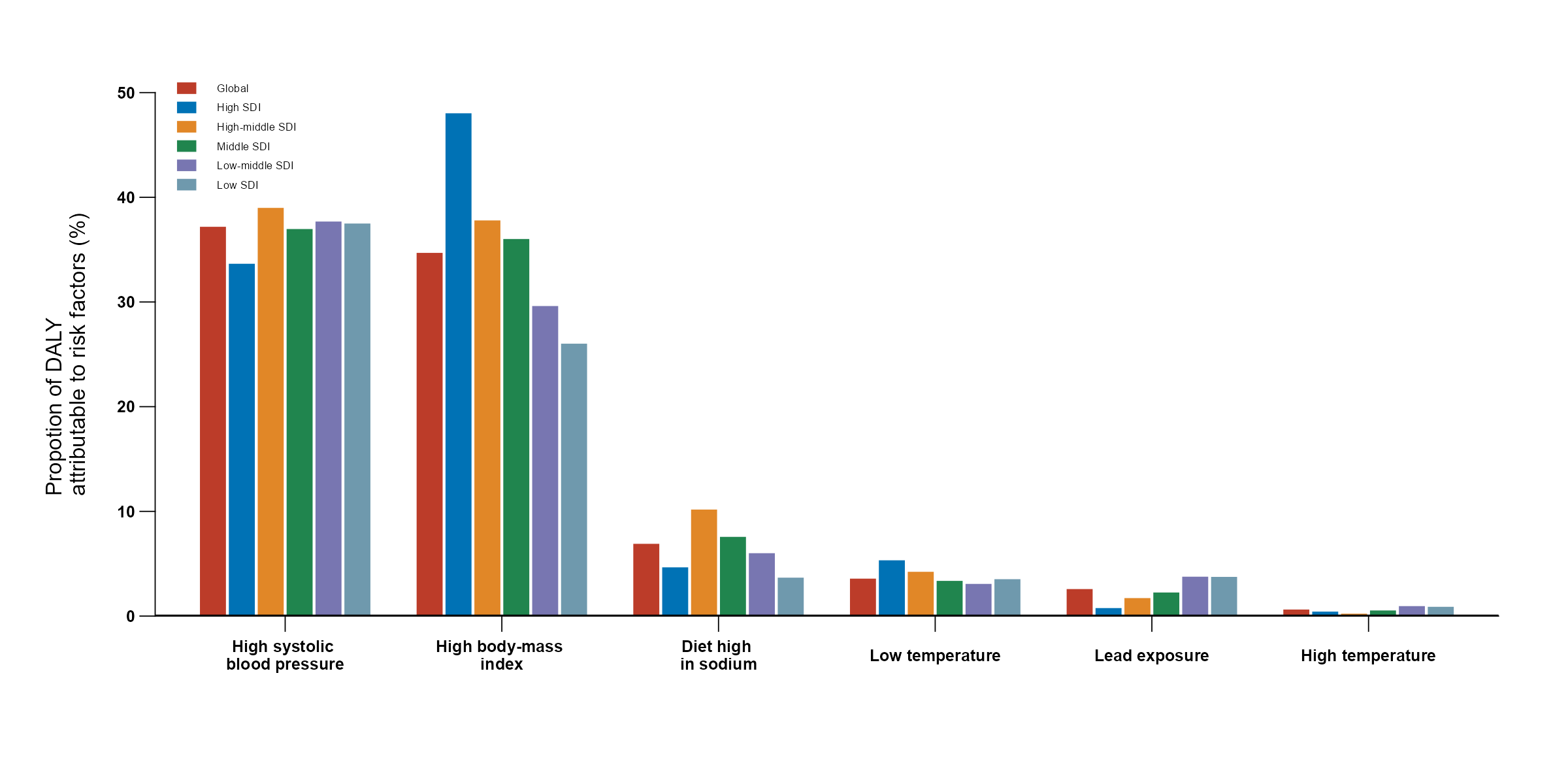

Supplement: Supplementary file 9 — Supplementary Figure S8. [file 41598_2023_47091_MOESM9_ESM.tif]

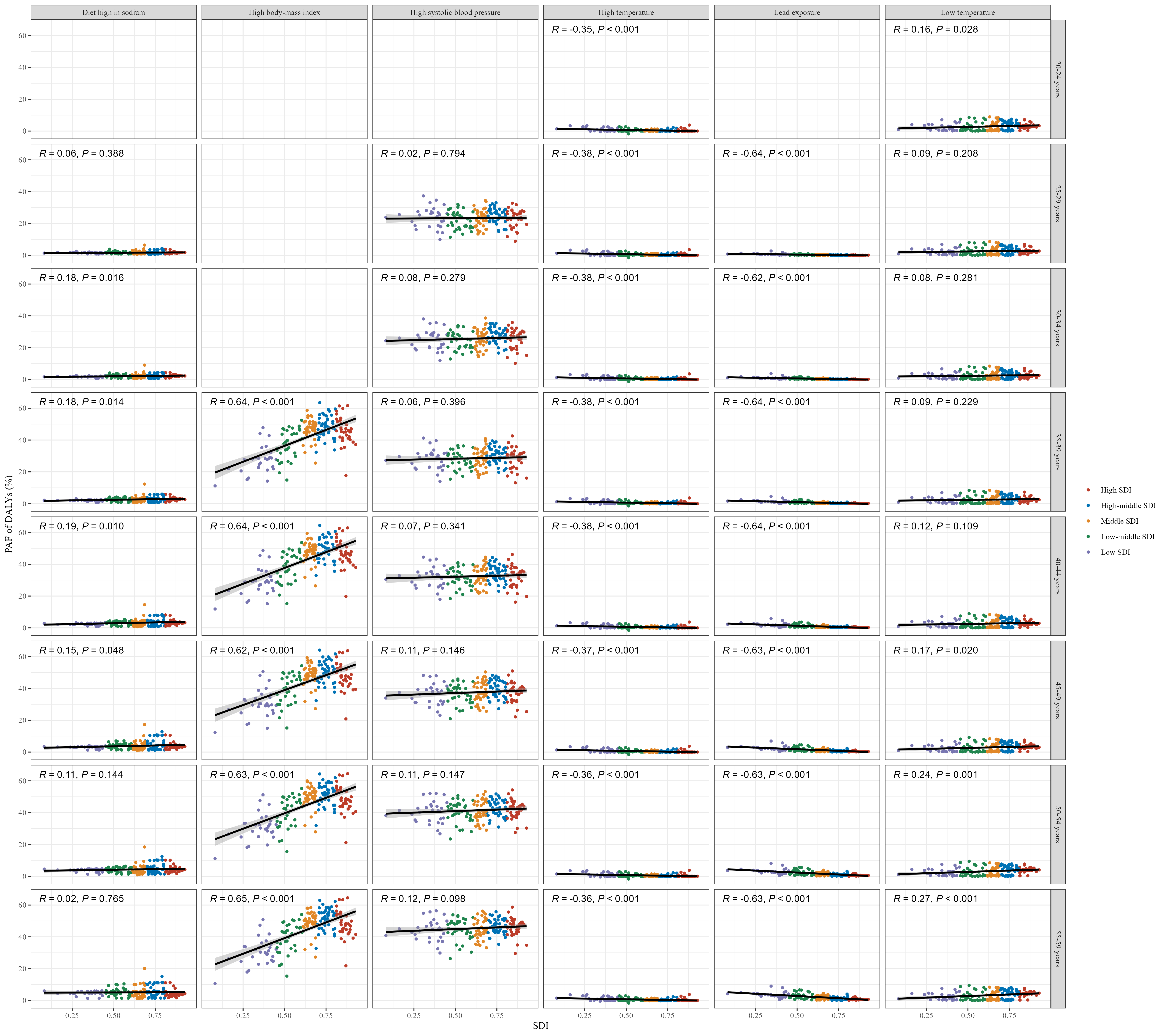

Supplement: Supplementary file 10 — Supplementary Figure S9. [file 41598_2023_47091_MOESM10_ESM.tif]
