# Supplementary material for: Global burden and influencing factors of chronic kidney disease due to type 2 diabetes in adults aged 20–59 years, 1990–2019
Source: Sci Rep. 2023 Nov 19;13:20234. doi: 10.1038/s41598-023-47091-y (PMC10658077; doi:10.1038/s41598-023-47091-y)
Supplement: Supplementary file 11 — Supplementary Information. [file 41598_2023_47091_MOESM11_ESM.docx]

**Supplementary Methods:**

**Age-standardized rate:**

Based on the Global Burden of Disease (GBD) 2019 study's world standard population, we estimated age-standardized rate (ASR)^1^ via the direct standardization method. The ASR (per 100 000) was then calculated using the following formula:

$$ASR=\frac{\sum_{i} d_{i}w_{i}}{y_{i}}$$

where $d_{i}$ represents the number of actual cases (or deaths) in the age group $i$, $y_{i}$ represents the overall person-years in the age group $i$, and $w_{i}$ represents the weight of age group $i$ in the world standard population.

**Average annual percent changes**

To evaluate changes over time in the incidence, mortality, and DALYs of CKD-T2D among the population aged 20-59 years globally from 1990 to 2019, we used the Joinpoint Regression Program software (version 4.9.1.0, National Cancer Institute, USA) to calculate the average annual percent changes (AAPCs) and their corresponding 95% uncertainty intervals^2^. The validity was tested using Monte Carlo simulations, starting with the minimum number of joinpoints and selecting the optimal model based on the sum of squared errors. If there was no statistically significant difference, the model with fewer joinpoints was selected. The AAPCs were calculated as the geometrically weighted average of the annual percent changes for each segment obtained from the joinpoint regression analysis^3^.

**Age-period-cohort effect:**

The age-period-cohort (APC) model, based on the Poisson distribution^4^, reflects the temporal trends in disease incidence or mortality across three dimensions: age, period, and cohort. However, the perfect collinearity among these three factors makes it difficult to estimate the true effects of each^5^. To address this issue, we fitted the two-factor and three-factor models and selected the most appropriate model for APC analysis to estimate the effects of age, period, and cohort on CKD-T2D incidence, mortality, and DALYs. In this study, the two-factor models were age-cohort and the three-factor model used the APC-IE method proposed by Yang et al.^6^. The smaller the values of Akaike's information criterion (AIC) and the Bayesian information criterion (BIC) with parameter penalty terms, the better the fit.

In the APC-IE model, we recoded age-specific rates into eight age groups with 5-year intervals (20-24, 25-29, ..., 55-59 years), six periods with 5-year intervals (1990-94, 1995-99, ..., 2015-19), and thirteen birth cohorts with 5-year intervals (1935-1939, 1940-1944, ..., 1995-1999) to estimate the net effects of age, period, and cohort on the incidence, mortality, and DALYs rates of CKD-T2D. The APC model can be expressed as follows^7^:

$$Y_{ij}=\mu+\alpha\times age_{i}+\beta\times period_{j}+\gamma\times cohort_{ij}+\epsilon$$

Where $Y_{ij}$ denotes the incidence, mortality, and DALYs of CKD-T2D in age group $i$, period $j$. $\alpha$, $\beta$, and $\gamma$ denoted the coefficients of age, period, and cohort, respectively. $\mu$ represented the intercept, and $\epsilon$ the residual. Then we calculated the relative risk (RR) based on the estimated coefficient, representing the RR of CKD-T2D incidence, mortality, or DALYs for a specific age, period, or birth cohort compared to the average level^8^. The APC-IE model was carried out using the Stata 16.0 (StataCorp, College Station, TX, United States).

**Decomposition analysis:**

Decomposition analysis is an analytical method that allows for determining the additive contribution of different factor differences to the overall effect difference^9^. To better understand the explanatory factors driving changes in the incidence, mortality, and DALYs rates of CKD-T2D among the global population aged 20-59 years between 1990 and 2019, we used the decomposition methodology proposed by Das Gupta to conduct a decomposition analysis of CKD-T2D DALYs by age structure, population growth, and epidemiological changes and referred to previous literature to guide our analysis^9-11^. The number of DALYs for each location is calculated using the following formula:

$${DALY}_{ay, py, ry}=\sum_{i=1}^{8} (a_{i, y}\times p_{y}\times r_{i, y})$$

where ${DALY}_{ay, py, ry}$ represents the DALYs based on the age group $a$, population $p$, and rate $r$ for a given year $y$. $a_{i, y}$ represents the age structure for the age group $i$ among the eight age groups in the given year $y$. $p_{y}$ represents the total population for the given year $y$. $r_{i, y}$ represents the DALY rate for the age group $i$ in the given year $y$. The contribution of each factor, including age structure, population growth, and epidemiological changes, to the DALY changes between 1990 and 2019 is determined by isolating the impact of one factor while holding the other factors constant. For example, we calculated the impact of age structure as follows:

$$a\_effect={[(DALY}_{a2019, p1990, r1990}{+DALY}_{a2019, p2019, r2019})/3+{(DALY}_{a2019, p1990, r2019}{+DALY}_{a2019, p2019, r1990})/6]-{[(DALY}_{a1990, p2019, r2019}{+DALY}_{a1990, p1990, r1990})/3+{(DALY}_{a1990, p2019, r1990}{+DALY}_{a1990, p1990, r2019})/6]$$

**References**

S1. Ahmad, O. B., Boschi-Pinto, C., Lopez, A. D., Murray, C. J. & Inoue, M. Age Standardization of Rates: A New WHO Standard. (2001).

S2. Kim, H. J., Fay, M. P., Feuer, E. J. & Midthune, D. N. Permutation tests for joinpoint regression with applications to cancer rates. *Stat Med* **19**, 335-351, doi:10.1002/(sici)1097-0258(20000215)19:3<335::aid-sim336>3.0.co;2-z (2000).

S3. Clegg, L. X., Hankey, B. F., Tiwari, R., Feuer, E. J. & Edwards, B. K. Estimating average annual per cent change in trend analysis. *Stat Med* **28**, 3670-3682, doi:10.1002/sim.3733 (2009).

S4. Mason, K. O., Mason, W. M., Winsborough, H. H., & Poole, W. Some Methodological Issues in Cohort Analysis of Archival Data. *Am Sociol Rev* **38**, 242-258 (1973).

S5. Yang, Y. Trends in U.S. adult chronic disease mortality, 1960-1999: age, period, and cohort variations. *Demography* **45**, 387-416, doi:10.1353/dem.0.0000 (2008).

S6. Yang, Y., Fu, W. J., & Land, K.C. *A Methodological Comparison of Age-Period-Cohort Models: Intrinsic Estimator and Conventional Generalized Linear Models*. Vol. 34 (Blackwell Publishing, 2004).

S7. Liu, X., Yu, C., Bi, Y. & Zhang, Z. J. Trends and age-period-cohort effect on incidence and mortality of prostate cancer from 1990 to 2017 in China. *Public Health* **172**, 70-80, doi:10.1016/j.puhe.2019.04.016 (2019).

S8. Keyes, K. M. & Miech, R. Age, period, and cohort effects in heavy episodic drinking in the US from 1985 to 2009. *Drug Alcohol Depend* **132**, 140-148, doi:10.1016/j.drugalcdep.2013.01.019 (2013).

S9. Xie, Y. *et al.* Analysis of the Global Burden of Disease study highlights the global, regional, and national trends of chronic kidney disease epidemiology from 1990 to 2016. *Kidney Int* **94**, 567-581, doi:10.1016/j.kint.2018.04.011 (2018).

S10. Das Gupta, P. Standardization and decomposition of rates from cross-classified data. *Genus* **50**, 171-196 (1994).

S11. Chevan, A. & Sutherland, M. Revisiting Das Gupta: refinement and extension of standardization and decomposition. *Demography* **46**, 429-449, doi:10.1353/dem.0.0060 (2009).
