# Supplementary material for: Global burden and influencing factors of chronic kidney disease due to type 2 diabetes in adults aged 20–59 years, 1990–2019
Source: Sci Rep. 2023 Nov 19;13:20234. doi: 10.1038/s41598-023-47091-y (PMC10658077; doi:10.1038/s41598-023-47091-y)
Supplement: Supplementary file 12 — Supplementary Table S1. [file 41598_2023_47091_MOESM12_ESM.docx]

Table S1 Numbers and ASRs per 100,000 in incidence, deaths, and DALYs for CKD-T2D in 2019, and percentage change from 1990 globally and by SDI quintile.

|  | **Incidence** | |  |  | **Deaths** | |  |  | **DALYs** | |  |
| --- | --- | --- | --- | --- | --- | --- | --- | --- | --- | --- | --- |
| **Location** | **Number, in thousands (95% UI)** | **ASRs per 100,000 (95% UI)** | **Percentage change from 1990** |  | **Number, in thousands (95% UI)** | **ASRs per 100,000 (95% UI)** | **Percentage change from 1990** |  | **Number, in thousands (95% UI)** | **ASRs per 100,000 (95% UI)** | **Percentage change from 1990** |
| **Global** | 2501.2 (2280.0 – 2740.8) | 30.3 (27.6 – 33.1) | 21.8% |  | 406.0 (328.4 – 485.0) | 5.2 (4.2 – 6.2) | 24.6% |  | 9870.4 (8114.8 – 11736.4) | 120.2 (99.2 – 142.9) | 18.2% |
| **High SDI**^a^ | 710.9 (648.7 – 775.2) | 37.7 (34.4 – 41.2) | 12.3% |  | 79.7 (61.6 – 99.5) | 3.7 (2.9 – 4.5) | 54.9 % |  | 1525.0 (1252.9 – 1803.4) | 80.8 (67.0 – 95.3) | 38.2% |
| **High-middle SDI**^a^ | 558.2 (506.3 – 613.4) | 27.1 (24.6 – 29.8) | 26.3% |  | 62.0 (49.9 – 75.2) | 3.1 (2.5 – 3.8) | 4.7% |  | 1495.2 (1229.3 – 1766.9) | 73.9 (61.1 – 87.1) | 1.4% |
| **Middle SDI**^a^ | 776.0 (705.3 – 850.2) | 30.5 (27.8 – 33.4) | 31.4% |  | 155.8 (126.6 – 185.5) | 7.0 (5.8 – 8.3) | 13.2% |  | 3984.3 (3268.7 – 4743.6) | 159.6 (132.1 – 188.2) | 11.5% |
| **Low-middle SDI**^a^ | 349.8 (315.7 – 387.0) | 25.3 (22.9 – 28.0) | 31.3% |  | 79.2 (62.0 – 96.5) | 6.4 (5.1 – 7.8) | 13.6% |  | 2104.0 (1648.6 – 2571.9) | 152.0 (119.8 – 184.3) | 12.2% |
| **Low SDI**^a^ | 105.0 (94.6 – 116.5) | 20.5 (18.5 – 22.7) | 25.3% |  | 28.9 (22.6 – 35.6) | 6.7 (5.3 – 8.2) | ﹣3.0% |  | 754.9 (588.0 – 928.1) | 146.4 (115.9 – 178.0) | ﹣2.2% |

ASRs: age standardized rates; CKD-T2D: chronic kidney disease due to type 2 diabetes; DALYs: disability adjusted life years; SDI: socio-demographic Index; UI: uncertainty intervals.

^a^Low SDI: SDI < 0.46; Low-middle SDI: 0.46 to 0.64; Middle SDI: 0.65 to 0.74; High-middle SDI: 0.75 to 0.85; High SDI: SDI > 0.85.
