# Supplementary material for: Global burden and influencing factors of chronic kidney disease due to type 2 diabetes in adults aged 20–59 years, 1990–2019
Source: Sci Rep. 2023 Nov 19;13:20234. doi: 10.1038/s41598-023-47091-y (PMC10658077; doi:10.1038/s41598-023-47091-y)
Supplement: Supplementary file 13 — Supplementary Table S2. [file 41598_2023_47091_MOESM13_ESM.docx]

**Table S2. The average annual percentage change of incidence, death and DALYs in CKD-T2D by age, from 1990 to 2019.**

| **Age group (years)** | **Incidence** | |  | **Deaths** | |  | **DALYs** | |
| --- | --- | --- | --- | --- | --- | --- | --- | --- |
|  | **AAPC** | **95% CI** |  | **AAPC** | **95% CI** |  | **AAPC** | **95% CI** |
| **ASR** | 1990-2005: 0.7 | (0.7– 0.7) |  | 1990-1995: 0.8 | (0.3– 1.2) |  | 1990-1995: 0.6 | (0.2– 1.0) |
|  | 2005-2011: 0.5 | (0.3– 0.6) |  | 1995-2005: 1.8 | (1.6– 1.9) |  | 1995-2005: 1.5 | (1.4– 1.7) |
|  | 2011-2019: 0.8 | (0.7– 0.8) |  | 2005-2019: 0.2 | (0.1– 0.3) |  | 2005-2019: 0.1 | (0.0– 0.1) |
| **20-24** | -1.2^*^ | (-1.3– -1.1) |  | -0.8^*^ | (-1.0– -0.5) |  | -0.8^*^ | (-1.0– -0.6) |
| **25-29** | -0.8^*^ | (-0.8– -0.7) |  | -0.8^*^ | (-1.2– -0.3) |  | -0.7^*^ | (-1.1– -0.4) |
| **30-34** | 0.0 | (-0.1– 0.1) |  | -0.7^*^ | (-1.1– -0.4) |  | -0.6^*^ | (-0.8– -0.3) |
| **35-39** | 0.5^*^ | (0.4– 0.6) |  | -0.5^*^ | (-0.6– -0.4) |  | -0.3^*^ | (-0.4– -0.2) |
| **40-44** | 0.7^*^ | (0.6– 0.7) |  | -0.1^*^ | (-0.3– 0.0) |  | 0.1 | (0.0– 0.2) |
| **45-49** | 0.6^*^ | (0.6– 0.7) |  | 0.0 | (-0.2– 0.2) |  | 0.2^*^ | (0.0– 0.3) |
| **50-54** | 0.7^*^ | (0.6– 0.7) |  | 0.2 | (-0.1– 0.6) |  | 0.4^*^ | (0.0– 0.7) |
| **55-59** | 0.8^*^ | (0.7– 0.8) |  | 0.4^*^ | (0.2– 0.6) |  | 0.5^*^ | (0.3– 0.7) |
| **all ages** | 2.0^*^ | (2.0– 2.0) |  | 2.2^*^ | (2.2– 2.3) |  | 1.8^*^ | (1.7– 1.9) |

AAPC: average annual percent change; ASR: age-standardized rate; CKD-T2D: chronic kidney disease due to type 2 diabetes; CI: confidence interval; DALY: disability adjusted life year.

^*^Significantly different from 0 at alpha = 0.05 (P < 0.05).
