# Supplementary material for: Global burden and influencing factors of chronic kidney disease due to type 2 diabetes in adults aged 20–59 years, 1990–2019
Source: Sci Rep. 2023 Nov 19;13:20234. doi: 10.1038/s41598-023-47091-y (PMC10658077; doi:10.1038/s41598-023-47091-y)
Supplement: Supplementary file 14 — Supplementary Table S3. [file 41598_2023_47091_MOESM14_ESM.docx]

**Table S3. Age, period, and cohort effects on the global incidence, death and DALYs relative risk of CKD-T2D.**

|  | **Incidence** | | |  | **Deaths** | | |  | **DALYs** | | |
| --- | --- | --- | --- | --- | --- | --- | --- | --- | --- | --- | --- |
|  | **RR** | **95% CI** | ***P-*value** |  | **RR** | **95% CI** | ***P-*value** |  | **RR** | **95% CI** | ***P-*value** |
| **Age (years)** | |  |  |  |  |  |  |  |  |  |  |
| 20-24 | 0.10 | (0.10-0.10) | 0.00 |  | 0.19 | (0.18-0.19) | 0.00 |  | 0.22 | (0.22-0.23) | 0.00 |
| 25-29 | 0.18 | (0.18-0.18) | 0.00 |  | 0.26 | (0.25-0.26) | 0.00 |  | 0.31 | (0.31-0.31) | 0.00 |
| 30-34 | 0.34 | (0.34-0.34) | 0.00 |  | 0.41 | (0.40-0.42) | 0.00 |  | 0.48 | (0.48-0.48) | 0.00 |
| 35-39 | 0.76 | (0.75-0.76) | 0.00 |  | 0.65 | (0.64-0.66) | 0.00 |  | 0.72 | (0.72-0.72) | 0.00 |
| 40-44 | 1.60 | (1.59-1.61) | 0.00 |  | 1.18 | (1.17-1.19) | 0.00 |  | 1.21 | (1.21-1.22) | 0.00 |
| 45-49 | 3.12 | (3.11-3.13) | 0.00 |  | 2.19 | (2.17-2.21) | 0.00 |  | 2.02 | (2.02-2.02) | 0.00 |
| 50-54 | 5.25 | (5.23-5.26) | 0.00 |  | 4.27 | (4.24-4.29) | 0.00 |  | 3.40 | (3.40-3.40) | 0.00 |
| 55-59 | 8.21 | (8.19-8.24) | 0.00 |  | 7.02 | (6.98-7.07) | 0.00 |  | 4.93 | (4.92-4.93) | 0.00 |
| **Period** |  |  |  |  |  |  |  |  |  |  |  |
| 1990 | 0.71 | (0.71-0.71) | 0.00 |  | 0.81 | (0.80-0.82) | 0.00 |  | 0.82 | (0.82-0.83) | 0.00 |
| 1995 | 0.80 | (0.80-0.80) | 0.00 |  | 0.88 | (0.87-0.88) | 0.00 |  | 0.88 | (0.88-0.88) | 0.00 |
| 2000 | 0.92 | (0.92-0.92) | 0.00 |  | 0.97 | (0.96-0.98) | 0.00 |  | 0.97 | (0.97-0.97) | 0.00 |
| 2005 | 1.06 | (1.06-1.06) | 0.00 |  | 1.04 | (1.04-1.05) | 0.00 |  | 1.04 | (1.04-1.04) | 0.00 |
| 2010 | 1.23 | (1.23-1.23) | 0.00 |  | 1.12 | (1.11-1.12) | 0.00 |  | 1.11 | (1.11-1.11) | 0.00 |
| 2015 | 1.46 | (1.46-1.47) | 0.00 |  | 1.24 | (1.23-1.25) | 0.00 |  | 1.23 | (1.22-1.23) | 0.00 |
| **Cohort** |  |  |  |  |  |  |  |  |  |  |  |
| 1935-1939 | 2.02 | (2.01-2.03) | 0.00 |  | 1.63 | (1.61-1.66) | 0.00 |  | 1.52 | (1.51-1.52) | 0.00 |
| 1940-1944 | 1.84 | (1.84-1.85) | 0.00 |  | 1.55 | (1.54-1.57) | 0.00 |  | 1.45 | (1.45-1.45) | 0.00 |
| 1945-1949 | 1.68 | (1.67-1.69) | 0.00 |  | 1.51 | (1.49-1.52) | 0.00 |  | 1.42 | (1.42-1.43) | 0.00 |
| 1950-1954 | 1.51 | (1.50-1.51) | 0.00 |  | 1.40 | (1.39-1.41) | 0.00 |  | 1.34 | (1.34-1.34) | 0.00 |
| 1955-1959 | 1.35 | (1.35-1.36) | 0.00 |  | 1.32 | (1.31-1.33) | 0.00 |  | 1.28 | (1.28-1.28) | 0.00 |
| 1960-1964 | 1.22 | (1.22-1.23) | 0.00 |  | 1.23 | (1.22-1.24) | 0.00 |  | 1.21 | (1.20-1.21) | 0.00 |
| 1965-1969 | 1.07 | (1.06-1.07) | 0.00 |  | 1.13 | (1.11-1.14) | 0.00 |  | 1.12 | (1.12-1.12) | 0.00 |
| 1970-1974 | 0.94 | (0.94-0.95) | 0.00 |  | 1.00 | (0.98-1.01) | 0.46 |  | 1.01 | (1.01-1.01) | 0.00 |
| 1975-1979 | 0.83 | (0.83-0.84) | 0.00 |  | 0.87 | (0.85-0.88) | 0.00 |  | 0.90 | (0.89-0.90) | 0.00 |
| 1980-1984 | 0.73 | (0.72-0.73) | 0.00 |  | 0.74 | (0.72-0.75) | 0.00 |  | 0.78 | (0.77-0.78) | 0.00 |
| 1985-1989 | 0.60 | (0.59-0.61) | 0.00 |  | 0.62 | (0.61-0.64) | 0.00 |  | 0.66 | (0.66-0.67) | 0.00 |
| 1990-1994 | 0.47 | (0.46-0.48) | 0.00 |  | 0.54 | (0.52-0.55) | 0.00 |  | 0.57 | (0.57-0.58) | 0.00 |
| 1995-1999 | 0.37 | (0.36-0.39) | 0.00 |  | 0.48 | (0.45-0.51) | 0.00 |  | 0.51 | (0.51-0.52) | 0.00 |

CKD-T2D: chronic kidney disease due to type 2 diabetes; CI: confidence interval; DALYs: disability adjusted life years; RR: relative risk [RR = exp(coefficient)].
