# Supplementary material for: Global burden and influencing factors of chronic kidney disease due to type 2 diabetes in adults aged 20–59 years, 1990–2019
Source: Sci Rep. 2023 Nov 19;13:20234. doi: 10.1038/s41598-023-47091-y (PMC10658077; doi:10.1038/s41598-023-47091-y)
Supplement: Supplementary file 15 — Supplementary Table S4. [file 41598_2023_47091_MOESM15_ESM.docx]

**Table S4. Changes in DALYs number according to population-level determinants from 1990 to 2019 globally and by SDI quintile.**

| **Location** | **Overall difference** | **Change due to population-level determinants**  **(% contribution to the total changes)** | | |
| --- | --- | --- | --- | --- |
|  |  | **Aging** | **Population growth** | **Epidemiologic change** |
| **Global** | 1,704,885.0 | 439381.2 (25.8%) | 1123149.7 (65.9%) | 142354.1 (8.3%) |
| **High SDI**^a^ | 132,903.3 | 45779.6 (34.5%) | 40008.8 (30.1%) | 47115 (35.5%) |
| **High-middle SDI**^a^ | 139,408.3 | 78162.2 (56.1%) | 114250.8 (82%) | -53004.7 (-38%) |
| **Middle SDI**^a^ | 789,861.5 | 294253.1 (37.2%) | 514683.9 (65.2%) | -19075.6 (-2.4%) |
| **Low-middle SDI**^a^ | 470,893.2 | 69013 (14.7%) | 356364.1 (75.7%) | 45516.2 (9.7%) |
| **Low SDI**^a^ | 170,245.2 | -8680.6 (-5.1%) | 182948.9 (107.5%) | -4023.1 (-2.4%) |

DALYs, disability-adjusted life-years; SDI, Sociodemographic Index.

^a^Low SDI: SDI < 0.46; Low-middle SDI: 0.46 to 0.64; Middle SDI: 0.65 to 0.74; High-middle SDI: 0.75 to 0.85; High SDI: SDI > 0.85.
