# Supplementary material for: Global burden and influencing factors of chronic kidney disease due to type 2 diabetes in adults aged 20–59 years, 1990–2019
Source: Sci Rep. 2023 Nov 19;13:20234. doi: 10.1038/s41598-023-47091-y (PMC10658077; doi:10.1038/s41598-023-47091-y)
Supplement: Supplementary file 16 — Supplementary Table S5. [file 41598_2023_47091_MOESM16_ESM.docx]

**Table S5. Attributable risk factors of DALYs in CKD-T2D.**

| **Categories** | **Risk factors** |
| --- | --- |
| behavioral risks | diet high in sodium |
| environmental/occupational risks | low temperature  high temperature  lead exposure |
| metabolic risks | high systolic blood pressure |
|  | high body-mass index |

CKD-T2D: chronic kidney disease due to type 2 diabetes; DALYs: disability adjusted life year
